# RhoA/Rock activation represents a new mechanism for inactivating Wnt/β-catenin signaling in the aging-associated bone loss

**DOI:** 10.1186/s13619-020-00071-3

**Published:** 2021-03-03

**Authors:** Wei Shi, Chengyun Xu, Ying Gong, Jirong Wang, Qianlei Ren, Ziyi Yan, Liu Mei, Chao Tang, Xing Ji, Xinhua Hu, Meiyu Qv, Musaddique Hussain, Ling-Hui Zeng, Ximei Wu

**Affiliations:** 1 Department of Pharmacology, Zhejiang University School of Medicine, 866 Yuhangtang Road, Hangzhou, 310058 China; 2 Department of Biology and Genetics, University of North Carolina-Chapel Hill, Chapel Hill, NC 27599 USA; 3 Department of Orthopeadic Surgery of Sir Run Run Shaw Hospital, Zhejiang University School of Medicine, Hangzhou, 310016 China; 4 Department of Pharmacology, Zhejiang University City College, 51 Huzhou Street, Hangzhou, 310015 China; 5 Translational Research Program in Pediatric Orthopaedics, The Children’s Hospital of Philadelphia, Philadelphia, PA 19104 USA

**Keywords:** RhoA, Rock, Wnt, β-Catenin, Limb bud, Bone

## Abstract

The Wnt/β-catenin signaling pathway appears to be particularly important for bone homeostasis, whereas nuclear accumulation of β-catenin requires the activation of Rac1, a member of the Rho small GTPase family. The aim of the present study was to investigate the role of RhoA/Rho kinase (Rock)-mediated Wnt/β-catenin signaling in the regulation of aging-associated bone loss. We find that Lrp5/6-dependent and Lrp5/6-independent RhoA/Rock activation by Wnt3a activates Jak1/2 to directly phosphorylate Gsk3β at Tyr216, resulting in Gsk3β activation and subsequent β-catenin destabilization. In line with these molecular events, *RhoA* loss- or gain-of-function in mouse embryonic limb bud ectoderms interacts genetically with *Dkk1* gain-of-function to rescue the severe limb truncation phenotypes or to phenocopy the deletion of *β-catenin*, respectively. Likewise, *RhoA* loss-of-function in pre-osteoblasts robustly increases bone formation while gain-of-function decreases it. Importantly, high RhoA/Rock activity closely correlates with Jak and Gsk3β activities but inversely correlates with β-catenin signaling activity in bone marrow mesenchymal stromal cells from elderly male humans and mice, whereas systemic inhibition of Rock therefore activates the β-catenin signaling to antagonize aging-associated bone loss. Taken together, these results identify RhoA/Rock-dependent Gsk3β activation and subsequent β-catenin destabilization as a hitherto uncharacterized mechanism controlling limb outgrowth and bone homeostasis.

**Supplementary Information:**

The online version contains supplementary material available at 10.1186/s13619-020-00071-3.

## Background

Wnt ligands activate both β-catenin-dependent and β-catenin-independent signaling pathways and play important roles in embryonic development and tissue homeostasis (van Amerongen and Nusse 2009). During Wnt/β-catenin signaling, the binding of Wnt to Frizzled (FZD) receptors and to the low-density lipoprotein receptor-related protein 5 or 6 (Lrp5/6) activates Dishevelled (Dvl) to stabilize cytosolic β-catenin. Upon its entry into the nucleus, β-catenin in turn activates the transcription of downstream target genes, including lymphoid enhancer-binding factor 1 (*Lef1*), cyclin D1 (*C.D1*), Dickkopf-1 (*Dkk1*), and *Axin2* (Huelsken and Birchmeier 2001; Wu et al. 2008). The secreted proteins Dkk1 and sclerostin (Sost) then antagonize the pathway by interfering with Lrp5/6-Wnt interactions, thereby providing a negative feedback mechanism that limits the range of Wnt signaling (Bafico et al. 2001; Wu et al. 2008).

Wnt/β-catenin signaling functions as a master regulator of limb development and bone formation; consequently, dysregulation of this pathway results in severe limb defects in mouse embryos and a failure to maintain bone mass in adult mice and humans (Barrow et al. 2003; Canalis et al. 2007; Karner and Long 2017). Notably, patients with osteoporosis or age-associated bone loss display a significantly low activity of Wnt/β-catenin signaling in bone marrow mesenchymal stromal cells (BMMSCs), primarily due to the high circulating levels of Dkk1 and Sost (Coulson et al. 2017; Hampson et al. 2013). Targeting the Wnt/β-catenin signaling therefore represents a promising therapeutic approach for treatment of osteoporosis or aging-associated bone loss (Jing et al. 2018; Manolagas and Almeida 2007).

Wnt signaling also occurs via two well-characterized β-catenin-independent pathways—the planar cell polarity (PCP) pathway and the Wnt/Ca^2+^ pathway—and both pathways inhibit β-catenin-dependent Wnt signaling (Nemeth et al. 2007; Yuzugullu et al. 2009). The PCP pathway is initiated by Wnt binding to Frizzled receptors and leads to the downstream activation of Rho family small GTPases (Fanto et al. 2000; Strutt et al. 1997). This family of GTPases includes RhoA, Rac1, and Cdc42, which serve as molecular switches with multiple roles that involve cycling between active GTP-bound and inactive GDP-bound forms (Bishop and Hall 2000). We have previously reported that nuclear accumulation of β-catenin requires the activation of Rac1. Specifically, we have shown that activation of c-Jun N-terminal kinase 2 (JNK2) by Rac1 results in phosphorylation of β-catenin on critical residues and promotes its nuclear translocation. We have also presented evidence that *Rac1* interacts genetically with *β-catenin* and *Dkk1* to control limb outgrowth in mouse embryos (Wu et al. 2008).

Despite this discovery of a role for Rho small GTPases in the β-catenin-dependent Wnt signaling pathway, the importance and biological relevance of RhoA in the regulation of Wnt/β-catenin signaling remain obscure. Here, we report that the regulation of aging-associated bone loss involves the destabilization of β-catenin by RhoA/Rho kinase (Rock) through activation of Janus kinase (Jak) and its direct phosphorylation of the Tyr216 residue of glycogen synthase kinase-3β (Gsk3β).

## Methods

### Antibodies, proteins, chemicals, and kits

Antibodies against Jak1, Jak2, Gsk3β, β-catenin, p-Ser9-Gsk3β, p-Ser552-β-catenin, p-Ser33/37/Thr41-β-catenin (p-Ser33-β-catenin), and non-p-Ser45-β-catenin (active β-catenin) were from Cell Signaling (Danvers, MA). Antibodies against p-Tyr1022-Jak1 and p-Tyr1007-Jak2 were purchased from Bioworld Technology (St. Louis Park, MN). Antibodies against Rock1, Rock2, Gsk3β, glyceraldehyde-3-phosphate dehydrogenase (GAPDH), β-actin, and Lamin B were obtained from Santa Cruz Biotechnology (Santa Cruz, CA). Antibody against alkaline phosphatase (Alp), bone sialoprotein (Bsp), Lef1, Nestin, p-Ser1366-Rock2, and p-Tyr216-Gsk3β and recombinant human active Jak2 and Gsk3β were from Abcam (Cambridge, UK). The IRDye 680, 700, and 800 s antibodies were from LI-COR Bioscience (Lincoln, NE). Alexa555- and Alexa488-conjugated secondary antibodies were from Molecular Probes (Eugene, OR). Recombinant mouse active Wnt3a (rWnt3a), Wnt5a (rWnt5a), and Dkk1 (rDkk1) were from StemRD (Burlingame, CA). Y27632 and Fasudil were from Selleckchem (Huston, TX) and Pyridone 6 was from Medchem Express (Monmouth Junction, NJ).

### Mouse strains, treatments, and embryos

*Msx2-Cre*, *Col1-Cre* (2.3 kb), *CAT-dnRhoA*^*+/−*^, *R26-Dkk1*^*+/−*^, and *β-catenin*^*f/f*^ mouse strains were as reported (Sun et al. 2000; Kobayashi et al. 2004; Wu et al. 2008; Barrow et al. 2003). The *RhoA*^*f/f*^ mouse strain was generated with CRISPR/Cas9- mediated genome-editing technique by Nanjing Biomedical Research Institute of Nanjing University (Nanjing, China) as described previously (Ma et al. 2014). The conditional *caRhoA* (G14V) knock-in mouse strain was generated by Cyagen Biosciences Inc. (Guangzhou, China) as described previously (Xu et al. 2019; Jackson et al. 2001). Mouse experiments were approved by the Zhejiang University Institutional Animal Care and Use Committee. Embryos were harvested for whole-mount skeleton preparation, whole-mount in situ hybridization, and limb bud sectioning and staining as previously described (Wu et al. 2008; Tu et al. 2007). Five-, 8-, and 10-month-old male Institute of Cancer Research (ICR) mice were purchased from Laboratory Animal Center of Zhejiang Province (Hangzhou, China). Two-, 5-, 8-, and 10-month-old male ICR mice were used for isolation of BMMSCs, and were also subjected to tibial sectioning. Eight-month-old male ICR mice were orally administrated with Fasudil once daily at 0 and 100 mg/kg for 2 months. Long bones were used for μCT examination or sectioning, whereas sera were used for ELISA assays of carboxyterminal collagen cross-links (CTX) (CUSABIO, Wuhan, China), tartrate-resistant acid phosphatase (Trap) (Immunodiagnostic Systems, Scottsdale, AZ), osteocalcin (OCN) (Immutopics, San Clemente, CA), and procollagen type 1 N-terminal propeptide (P1NP) (MyBioSource, San Diego, CA). We used μCT (μCT 40, Scanco Medical) for three-dimensional reconstruction and quantification of bone parameters, and reconstructed each image from one hundred 16-μm slices immediately below the growth plate, with a threshold of 200 (Gong et al. 2014; Hu et al. 2018).

### Isolation of human BMMSCs

Bone marrows were harvested from four elderly men (74-, 78-, 82-, and 84-year-old) who suffered from femoral neck fracture and underwent the total hip replacement surgery and three young men (28-, 32-, 33-year-old) who suffered from traumatic fracture and underwent the internal fixation surgery in the Department of Orthopeadic Surgery, Sir Run Run Shaw Hospital, Zhejiang University School of Medicine. The young men were hurt in accidents and had no osteoporosis or other diagnosed diseases. The elderly men were satisfied with the diagnosis of osteoporosis according to the commonly accepted WHO definitions and criteria, and had no other diagnosed diseases. Bone marrows were used for preparation of smears or isolation of BMMSCs by the Human Marrow Lymphocyte Medium Kit (Sangon Biotech, Shanghai, China). For quantification of bone marrow smear staining, phospho-ROCK2^+^, p-Ser33-β-CATENIN^+^, active β-CATENIN^+^, p-JAK1/2^+^, or p-Tyr216-GSK3β^+^ cells were raised from every 200 NESTIN^+^ cells.

### Isolation and culture of mouse BMMSCs

Mouse BMMSCs were isolated by a MagCellect Mouse Mesenchymal Stem Cell Isolation Kit (R&D Systems, Minneapolis, MN) as described previously (Gong et al. 2014). BMMSCs were cultured in the Roswell Park Memorial Institute (RPMI) medium supplemented with 10% heat-inactivated fetal calf serum (FCS) (Invitrogen, Carlsbad, CA), 100 IU/ml penicillin, and 100 mg/ml streptomycin (Invitrogen). Culture medium was changed at day 3 to remove the nonadherent cells. Cells were grown for 3 ~ 4 weeks until almost confluency. Adherent cells were then detached and replated using a 1:3 dilution until passage 2. Subsequent passaging and seeding of the cells were performed at a density of 5000 cells/cm^2^. BMMSCs were used for experiments after 4 ~ 7 passages.

### Cell cultures

Primary mouse calvarial osteoblasts (PMCOBs) were isolated from neonatal mice at postnatal day (P) 2 ~ 5, and expanded and maintained as described previously (Gong et al. 2014; Hu et al. 2018; Chen et al., 2017). C3H10T1/2 cells, HEK293T cells, and L929 cells were obtained from the American Type Culture Collection (ATCC), authenticated using short tandem repeat (STR) profiling, and maintained as described previously (Gong et al. 2014; Hu et al. 2018; Chen et al., 2017). No cell line used in this study was found in the database of commonly misidentified cell lines that is maintained by ICLAC and NCBI Biosample. Cells were passaged for no more than 6 months and routinely assayed for mycoplasma contamination.

### Transfection, lentiviral infection, dual-luciferase and rho activation assays

Constructs expressing the interest genes were cloned by using specific primers (Table S1), and gene-specific siRNAs were synthesized by Sangon Biotech (Shanghai, China) (Table S2). Transient transfection and *Lef1-luciferase* reporter assay were performed in C3H10T1/2 cells by using Lipofectamine 2000 (Invitrogen) as described previously (Gong et al. 2014; Hu et al. 2018). Dual-luciferase assay was performed according to the manufacturer’s instructions (Promega, Madison, WI). The firefly luciferase activities were normalized to Renilla luciferase levels. Infection and preparation of lentiviruses expressing the interest genes or shRNAs were as described previously (Table S3) (Hu et al. 2018). Assays for RhoA, Rac1 and Cdc42 activation were performed by using Active GTPase Pull-Down and Detection Kits (Pierce Biotechnology, Rockford, IL) as described previously (Wu et al. 2008). Total RhoA, Rac1, and Cdc42 were used as internal controls for their active forms (GTP binding form).

### Western blotting, immunoprecipitation, and in vitro phosphorylation assays

The cytosolic and nuclear fractions of cells were prepared as previously described (Wu et al. 2008; Gong et al. 2014). Immunoprecipitation and western blot analyses were performed using standard protocols as previously described (Wu et al. 2008). The in vitro kinase assay to assess the phosphorylation of GSK3β by JAK2 was performed as previously described (Wu et al. 2008). To validate JAK2 as a kinase phosphorylating GSK3β at Y216, recombinant human active JAK2 (Abcam, ab42621) and human GSK3β (Abcam, ab63193) were used in the in vitro kinase assay. 30 ng of GSK3β was incubated with 50 μl kinase assay buffer (Cell Signaling) in the presence or absence of 30 ng active JAK2 and 2 mM ATP at 37 °C for 30 min. The reactive mixture was subjected to western analyses with antibodies recognizing p-Tyr216-GSK3β, GSK3β, and JAK2. For western, GAPDH or β-actin was used as the internal standard of total target proteins, and phosphorylated proteins were normalized to total proteins, respectively. Immunoreactive bands from duplicates or triplicates were quantified by ImageJ, and the mean intensity from first band was set to 1.

### Osteoblast differentiation assay and quantitative RT-PCR

C3H10T1/2 cells or PMCOBs were subjected to Alp activity and formation of mineralized nodule assays as previously described (Wu et al. 2008; Gong et al. 2014). Alp activity was expressed as nanomoles of p-nitrophenol formed per minute per milligram of protein. For mineralization assays, cells were incubated in the regular growth medium containing 10% FCS, 50 mg/ml ascorbic acid, 100 nM dexamethasone, and 10 μM β-glycerophosphate (β-GP) for 21 days. Cells were stained with Alizarin-Red S (Sigma) and then extracted with 10% cetylpyridinium chloride (Sigma) for quantification as previously described (Chen et al., 2017). RNA was extracted from cultured cells or femurs by using TRIzol reagent (Takara Biotechnology). Messenger RNAs of *Alp*, *Bsp*, *Runx2*, *Lef1*, *Cyclin D1*, and *Axin2* were assayed as previously described (Gong et al. 2014). GAPDH or β-actin was included as an internal control and the relative values of mRNA species of interest were calculated by the 2^−ΔΔCt^ method.

### Histology, immunostaining, and dynamic bone histomorphometry

Mice were euthanized by CO_2_ asphyxiation, and tissues were fixed in 4% paraformaldehyde followed by decalcification and processing. Selected bones were embedded and sectioned for H&E staining, Trap staining, and immunostaining as described previously (Gong et al. 2014; Hu et al. 2018; Chen et al., 2017). The quantitative histomorphometry was performed using the ImageJ (NIH) and Osteomeasure Analysis System (Osteometrics, Decatur, GA) according to the recommendations of the Histomorphometry Nomenclature Committee of the American Society of Bone and Mineral Research (Dempster et al. 2013). Every 200 Nestin^+^ cells were manually scored as either positive or negative staining for phospho-Rock2, p-Jak1/2, p-Ser33-β-catenin, active β-catenin. To determine the positively stained areas surrounding Alp^+^ or bone surface areas, five fields of view per mouse per antibody were manually scored as either positive or negative staining. Dynamic bone histomorphometry was performed as previously (Chen et al., 2017). 8-μm-thick longitudinal tibial sections were prepared. Unstained sections were used for assessment of mineral apposition rate (MAR, μm/day), whereas new bone formation was assessed by fluorescence microscopy of Calcein (green). Histomorphometric analysis was performed by a blinded observer using BioQuant OSTEO 2010 software (BioQuant Image Analysis Corporation, Nashville, TN).

### Statistics

Numerical data was expressed as means ± SD or SEM, and statistical analysis was performed by using the SPSS statistical package (IBM, North Castle, NY). We determined significance by Student’s *t* test or one-way ANOVA and Tukey–Kramer multiple comparisons test. Statistical significance was assessed at levels of *p* < 0.05 and *p* < 0.01. Experiments were independently repeated and the representative experiments are shown.

## Results

### RhoA/Rock activation by Wnt3a constrains β-catenin signaling

**Fig. 1:**
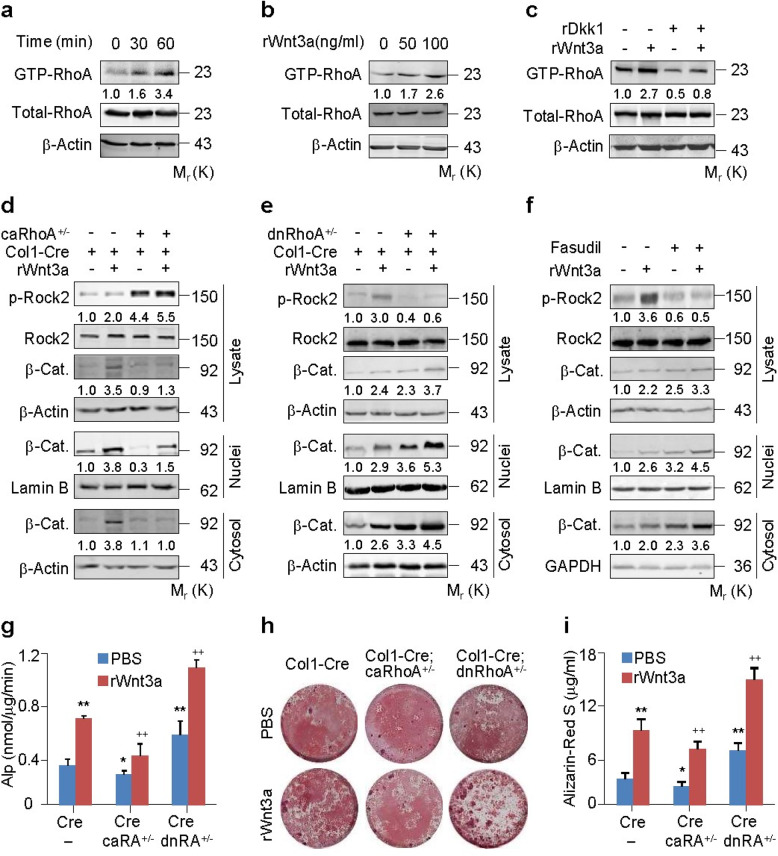
RhoA/Rock constraints Wnt/β-catenin signaling and osteoblastic differentiation. **a-c** RhoA activation assays in primary murine calvarial osteoblasts (PMCOBs) stimulated with rWnt3a at 100 ng/ml or the indicated concentrations for the indicated times or 60 min in the presence or absence of recombinant Dkk1 (rDkk1) at 100 ng/ml. **d**, **e** Western analyses of β-catenin in cytosolic and nuclear fractions of PMCOBs with the indicated genotypes of *Col1-Cre* (*Cre*), *Col1-Cre;caRhoA*^*+/−*^ (*Cre;caRhoA*^*+/−*^) or *Col1-Cre;dnRhoA*^*+/−*^ (*Cre;dnRhoA*^*+/−*^), and in the presence or absence of rWnt3a for 3 h. **f** Western analyses of β-catenin (β-cat) in cytosolic and nuclear fractions of PMCOBs treated with or without Fasudil at 20 μM and stimulated with or without rWnt3a for 3 h. **g-i** Alp activity and mineralization nodule formation assays and their quantification in PMCOBs with the indicated genotypes and stimulated with or without rWnt3a at 100 ng/ml for 48 h and 21 d, respectively. Mean ± SEM, ^*^p < 0.05, ^**,++^*p* < 0.01, *n* = 4, Tukey-Kramer multiple comparisons test

To determine the role of RhoA/Rock in Wnt/β-catenin signaling, we used an established binding assay to examine the levels of GTP-bound (active) form in primary murine calvarial osteoblasts (PMCOBs). Stimulation with recombinant mouse Wnt3a resulted in time-dependent and dose-dependent increases in the GTP-RhoA levels in PMCOBs (Fig. 1a, b). Activation of RhoA by Wnt3a was confirmed in several cell lines, including C3H10T1/2 cells, a cell line of mouse embryonic fibroblasts, HEK293T cells, and L929 cells (Fig. S1A-C). The potential involvement of Lrp5/6 was assessed by treating the PMCOBs with recombinant mouse Dkk1 protein. A Wnt3a treatment increased the GTP-RhoA levels regardless of the presence or absence of Dkk1; however, Dkk1 inhibited both the basal and Wnt3a-induced increases in GTP-RhoA levels (Fig. 1c). Rac1 or Cdc42 antagonizes RhoA signaling in many cell types, and Rac1 activation by Wnt3a enhances β-catenin signaling (Wan et al. 2013; Wu et al. 2008). For these reasons, we also examined the potential involvement of Rac1/Cdc42 in the RhoA activation in response to Wnt3a. We found that Wnt3a activated the different small GTPases concurrently and independently in C3H10T1/2 cells (Fig. S1D-F). Thus, Wnt3a activates RhoA in both an Lrp5/6-dependent and Lrp5/6-independent manner, but in a Rac1- or Cdc42-independent manner.

We then explored the exact role of RhoA in Wnt/β-catenin signaling by crossing mice that conditionally expressed the dominant negative form of RhoA (dnRhoA, T19N) or the constitutively active form of RhoA (caRhoA, G14V) with the mice expressing Cre recombinase under the control of the 2.3-kb mouse *collagen1α1* (*Col1*) promoter. This generated *Col1-Cre;dnRhoA*^*+/−*^ or *Col1-Cre;caRhoA*^*+/−*^ neonates and their control littermates. We then isolated PMCOBs from these mouse strains (P2 ~ P5) and conducted western blot analyses to identify the cytosolic versus nuclear fractions of β-catenin. Activation of RhoA in the PMCOBs from the *Col1-Cre;caRhoA*^*+/−*^ neonates and its inactivation in PMCOBs from *Col1-Cre;dnRhoA*^*+/−*^ neonates were validated by determining the p-Rock2 levels (Fig. 1d, e). Activation (inactivation) of RhoA in PMCOBs slightly reduced (increased) the β-catenin in the whole-cell lysate as well as in the cytosolic or nuclear fraction under basal conditions, whereas activation (inactivation) of RhoA notably suppressed (enhanced) the β-catenin accumulation in these cell fractions in response to Wnt3a (Fig. 1d, e). Likewise, the mRNA levels of *Lef1*, *Cyclin D1*, and *Axin2*, which are targets of Wnt/β-catenin signaling, were significantly decreased in PMCOBs of *Col1-Cre;caRhoA*^*+/−*^ neonates, or increased in PMCOBs of *Col1-Cre;dnRhoA*^*+/−*^ neonates, in either the presence or absence of Wnt3a (Fig. S2A, B). We also confirmed the role of RhoA in Wnt/β-catenin signaling by overexpressing either caRhoA or RhoA siRNA (RhoA-si) in C3H10T1/2 cells (Fig. S2D, E). All these findings are consistent with previous studies showing that inactivation of RhoA increases both the cytosolic and the nuclear levels of β-catenin (Peng et al. 2011; Shusterman et al. 2014; Miyagi et al. 2015). Thus, RhoA activation in response to Wnt3a constrains Wnt/β-catenin signaling by decreasing both the cytosolic and nuclear levels of β-catenin.

**Fig. 2:**
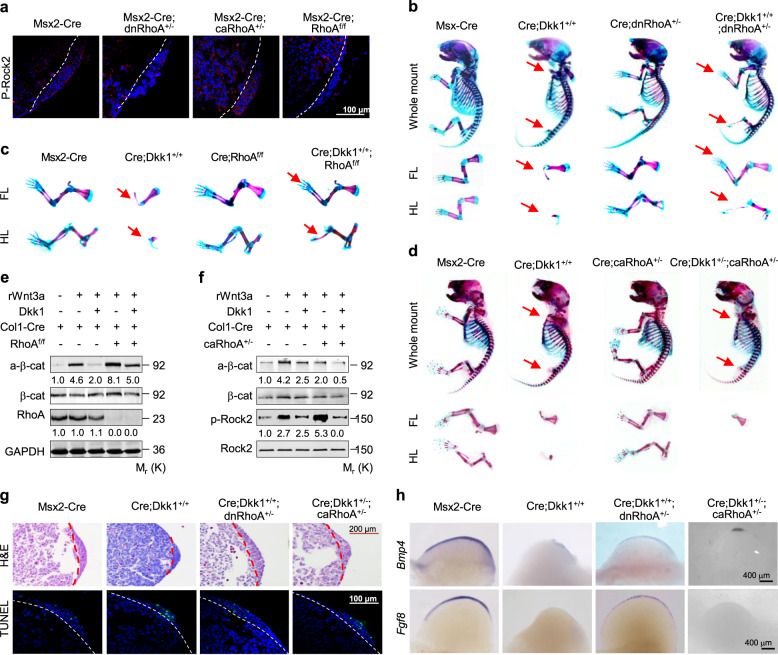
*RhoA* interacts genetically with *Dkk1* in the limb bud ectoderm of mouse embryos. **a** Representative immunostaining for p-Rock2 in the E10.5 limb buds with the indicated genotypes. White dot lines separate the apical ectodermal ridge (AER) from the zone of polarizing activity (ZPA). **b-d** Skeletons and/or limbs of E16.5 embryos with the indicated genotypes. **e**, **f** Western analyses in PMCOBs with the indicated genotypes and stimulated with or without rWnt3a at 100 ng/ml for 1 h in the presence or absence of rDkk1 at 100 ng/ml. Phosphorylated proteins were normalized to their total amounts, respectively. **g**, **h** H&E and TUNEL staining in E10.5 forelimb bud sections (**g**) and whole-mount in situ hybridization of E10.5 forelimb buds (**h**). Dot lines separate the AER from the ZPA. Ventral view for all limb buds, anterior to the lower and posterior to the upper. FL: forelimb, HL: hindlimb

Rock1/2 is the most extensively studied effectors of RhoA and is involved in many aspects of cell functions (Bishop and Hall 2000). We treated the wild type PMCOBs with a Rock2 inhibitor, Fasudil, to test whether Rock behaves essentially the same as *RhoA* loss-of function in the regulation of Wnt/β-catenin signaling. Fasudil treatment of PMCOBs slightly induced β-catenin expression in whole-cell lysates and in the cytosolic or nuclear fractions under basal conditions or robustly induced this expression in response to Wnt3a (Fig. 2f). Likewise, Fasudil treatment of PMCOBs consistently enhanced the mRNA levels of *Lef1*, *Cyclin D1*, and *Axin2* (Fig. S2C). The specific role of Rock1/2 in Wnt/β-catenin signaling was confirmed in C3H10T1/2 cells using Rock1/2-si, a constitutively active form of Rock2 (caRock2), or another Rock1/2 inhibitor Y27632 (Fig. S3A-C). Thus, Rock1/2 functions as a downstream effector of RhoA in the regulation of Wnt/β-catenin signaling.

Wnt/β-catenin signaling is the master regulator of BMMSC differentiation toward the osteoblastic lineage. We assessed the biological relevance of RhoA/Rock in the regulation of Wnt/β-catenin signaling by testing PMCOBs from *Col1-Cre*, *Col1-Cre;caRhoA*^*+/−*^, and *Col1-Cre;dnRhoA*^*+/−*^ neonates for their ability to undergo osteoblastic differentiation in response to Wnt3a. Activation of RhoA in PMCOBs significantly reduced the activity of alkaline phosphatase (Alp), an osteoblast marker, and the formation of mineralized nodules in either the presence or absence of Wnt3a, while inactivation enhanced these responses (Fig. 1g-i). These findings are consistent with previous studies showing that Rock negatively regulates osteoblastic differentiation and bone formation (Ohnaka et al. 2001; Kanazawa et al. 2009, 2010). Thus, RhoA/Rock negates Wnt/β-catenin signaling, thereby suppressing osteoblastic differentiation.

### *RhoA* interacts genetically with *Dkk1* in the AER

We established the genetic role of RhoA in the regulation of Wnt/β-catenin signaling using *RhoA* loss-of-function or gain-of-function at the apical ectodermal ridge (AER) of the mouse embryonic limb bud, where Wnt signaling is critical for limb outgrowth via β-catenin (Barrow et al. 2003). Specifically, we generated *Msx2-Cre*, *Msx2-Cre;dnRhoA*^*+/−*^, *Msx2-Cre;caRhoA*^*+/−*^, and *Msx2-Cre;RhoA*^*f/f*^ mouse embryos, taking advantage of the fact that *Msx2-Cre* is expressed in the limb bud ectoderm that gives rise to the AER (Sun et al. 2000). We used immunostaining for p-Rock2 to validate the genetic modifications in the E10.5 limb buds and found a significantly abolished or diminished p-Rock2 expression in the AER of the *Msx2-Cre;dnRhoA*^*+/−*^ or *Msx2-Cre;RhoA*^*f/f*^ embryos, respectively, but a robust enhancement of expression in the AER of the *Msx2-Cre;caRhoA*^*+/−*^ embryos (Fig. 2a).

The *Msx2-Cre;dnRhoA*^*+/−*^, *Msx2-Cre;caRhoA*^*+/−*^, and *Msx2-Cre;RhoA*^*f/f*^ embryos at E16.5 displayed similarly long and normally structured forelimbs and hindlimbs, with autopods, zeugopods, and stylopods, when compared with their control littermates (Fig. 2b-d). We therefore generated *Msx2-Cre;dnRhoA*^*+/−*^*;Dkk1*^*+/+*^ embryos by crossing the female *dnRhoA*^*+/−*^*;Dkk1*^*+/−*^ mice with male *Msx2-Cre;Dkk1*^*+/−*^ mice. The *Msx2-Cre;Dkk1*^*+/+*^ embryos at E16.5 lacked all hindlimb structures, including the autopods, zeugopods, and stylopods, and displayed truncations at various levels in the forelimbs (Fig. 2b-d), consistent with our previous findings (Wu et al. 2008). Remarkably, the *Msx2-Cre;dnRhoA*^*+/−*^*;Dkk1*^*+/+*^ embryos (6/6) exhibited normal forelimbs and varying lengths of hindlimbs but entirely lacked autopods (Fig. 2b). To confirm the specificity of *dnRhoA*, we then crossed female *RhoA*^*f/f*^*;Dkk1*^*+/−*^ mice with male *Msx2-Cre;RhoA*^*f/+*^*;Dkk1*^*+/−*^ mice to generate *Msx2-Cre;RhoA*^*f/f*^*;Dkk1*^*+/+*^ embryos that had a conditional knockout of *RhoA* and overexpression of *Dkk1* in the limb bud ectoderms. The *Msx2-Cre;RhoA*^*f/f*^*;Dkk1*^*+/+*^ embryos (4/4) exhibited the same limb phenotypes as the *Msx2-Cre;dnRhoA*^*+/−*^*;Dkk1*^*+/+*^ embryos, including the varying hindlimb length and lack of autopods (Fig. 2c). Thus, *RhoA* loss-of-function was sufficient to rescue the limb defects caused by *Dkk1* overexpression in the limb bud ectoderm.

We explored a specific role for RhoA in Wnt/β-catenin signaling by determining whether the two molecules genetically interact in the AER. To do this, we crossed *caRhoA*^*+/−*^ and *Msx2-Cre;Dkk1*^*+/−*^ mice to generate embryos that expressed both *caRhoA* and *Dkk1* in the AER. The resulting embryos with *Msx2-Cre* and one copy of either the *Dkk1* or *caRhoA* allele (*Msx2-Cre;Dkk1*^*+/−*^ or *Msx2-Cre;caRhoA*^*+/−*^) did not show any obvious limb phenotype changes; however, the double-heterozygous embryos (*Msx2-Cre;Dkk1*^*+/−*^*;caRhoA*^*+/−*^) (6/6) all failed to develop hindlimbs and nearly all the forelimbs (10/12 from 6 embryos) lacked structures distal to the scapula; these phenotypes are identical to those of the *Msx2-Cre;Dkk1*^*+/+*^ or *Msx2-Cre;β-catenin*^*n/f*^ embryos (Fig. 2d). One embryo was an exception and lacked forelimb structures distal to the humerus. Overall, *RhoA* was confirmed to interact genetically with *Dkk1* in regulating Wnt/β-catenin signaling and limb outgrowth.

### RhoA regulation of the limb outgrowth depends on Wnt/β-catenin signaling

We examined whether the effect of RhoA on limb bud outgrowth depends on β-catenin by crossing female *Msx2Cre;β-catenin*^*f/+*^ with male *dnRhoA*^*+/−*^*;β-catenin*^*f/f*^ mice to generate *Msx2Cre;β-catenin*^*n/f*^*;dnRhoA*^*+/−*^ embryos, taking advantage of the maternal germline transmission of *Msx2-Cre*. Unlike the *Msx2-Cre;dnRhoA*^*+/−*^*;Dkk1*^*+/+*^ embryos, the *Msx2-Cre;dnRhoA*^*+/−*^*;β-catenin*^*n/f*^ embryos (6/6) at E16.5 still lacked all hindlimb structures and exhibited truncations at various levels in the forelimbs; these defects were identical to those in the *Msx2-Cre;β-catenin*^*n/f*^ embryos (Fig. S4A). We also generated the *Msx2-Cre;Dkk1*^*+/+*^*;β-catenin*^*n/f*^ and *Msx2-Cre;dnRhoA*^*+/−*^*;Dkk1*^*+/+*^*;β-catenin*^*n/f*^ embryos at E16.5 to determine whether *dnRhoA* would restore the *Msx2-Cre;Dkk1*^*+/+*^phenotype in the absence of *β-catenin*. As expected, the *Msx2-Cre;Dkk1*^*+/+*^*;β-catenin*^*n/f*^ embryos lacked both forelimbs and hindlimbs and displayed the more severe limb phenotypes than either the *Msx2-Cre;Dkk1*^*+/+*^or *Msx2-Cre;β-catenin*^*n/f*^ embryos. However, However, expression of *dnRhoA* in AER did not result in any rescue of the limb phenotypes of *Msx2-Cre;Dkk1*^*+/+*^*;β-catenin*^*n/f*^ embryos (Fig. S4B). Thus, RhoA genetically regulates limb outgrowth, depending on β-catenin signaling.

We then determined the molecular events of *RhoA* loss-of-function or gain-of function in regulating the limb phenotypes caused by *Dkk*-overexpression in AER by performing western blot assays in PMCOBs isolated from *Col-Cre*, *Col-Cre;RhoA*^*f/f*^ or *Col-Cre;caRhoA*^*+/−*^ neonates. Wnt3a significantly increased the levels of active β-catenin, and Dkk1 robustly diminished this induction by Wnt3a in *Col-Cre* PMCOBs. Importantly, deletion of *RhoA* robustly increased the levels of active β-catenin and activation of RhoA robustly decreased those levels, whereas deletion of *RhoA* significantly restored and activation of RhoA significantly potentiated the Dkk1-negated active β-catenin in the *Col-Cre;RhoA*^*f/f*^ and *Col-Cre;caRhoA*^*+/−*^ PMCOBs in response to Wnt3a (Fig. 2e, f). Thus, *RhoA* loss-of-function was sufficient to restore, and gain-of-function was sufficient to potentiate, the Dkk1-reduced β-catenin levels.

We sought to gain further insights into the role of RhoA in the limb bud ectoderm by performing histological and TUNEL assays and in situ hybridization in E10.5 embryos. Consistent with the limb truncation phenotype, the *Msx2-Cre;Dkk1*^*+/+*^ embryos displayed noticeably smaller forelimbs and hindlimb buds than their control littermates; however, overexpression of *dnRhoA* in the AER of *Msx2-Cre;Dkk1*^*+/+*^ embryos significantly increased the sizes of both fore- and hindlimb buds, while overexpression of *caRhoA* in the AER of *Msx2-Cre;Dkk1*^*+/−*^ embryos significantly decreased these bud sizes (Fig. 2g and Fig. S5A). Likewise, Likewise, cell apoptosis in the AER was significantly lower in *Msx2-Cre;Dkk1*^+/+^*;dnRhoA*^*+/−*^ embryos than in *Msx2-Cre;Dkk1*^+/+^ embryos, whereas cell apoptosis was similar in the AER of the *Msx2-Cre;Dkk1*^+/−^*;caRhoA*^*+/−*^ and *Msx2-Cre;Dkk1*^*+/+*^ embryos (Fig. 2g and Fig. S5A, C).

The striking morphological alterations caused by overexpression of *dnRhoA* in *Msx2-Cre;Dkk1*^*+/+*^ embryos or of *caRhoA* in *Msx2-Cre;Dkk1*^*+/−*^ embryos prompted us to examine the potential regulation of RhoA at the molecular level. We performed in situ hybridization for fibroblast growth factor 8 (*Fgf8*) in the AER and bone morphogenetic protein 4 (*Bmp4*) in the distal ventral ectoderm; both of these are target genes of Wnt/β-catenin signaling. As expected, the *Msx2-Cre;Dkk1*^*+/+*^ embryos expressed only residual *Bmp4* and no *Fgf8* in the forelimb of E10.5 (34–35 somites) embryos (Fig. 2h and Fig. S5B, D). By contrast, overexpression of *dnRhoA* largely restored the expression of *Fgf8* and *Bmp4* in the *Msx2-Cre;Dkk1*^*+/+*^ background, whereas overexpression of *caRhoA* robustly diminished the expression of both *Fgf8* and *Bmp4* in the *Msx2-Cre;Dkk1*^*+/−*^ background (Fig. 2h and Fig. S5B, D). Both morphologically and molecularly, RhoA interacts with Wnt/β-catenin signaling to regulate the limb truncation phenotype caused by *Dkk1* overexpression in the AER.

### RhoA/Rock activates Gsk3β to destabilize β-catenin

We next examined the downstream events of RhoA/Rock in the regulation of Wnt/β-catenin signaling. Both protein kinase B (Akt) and Gsk3β have been suggested to function as effectors of RhoA/Rock signaling and both are involved in the phosphorylation of β-catenin (Papakonstanti et al. 2007; Kim et al. 2017). Therefore, we performed western blot analyses with antibodies specific for p-Ser552-β-catenin (an Akt phosphorylation site) and p-Ser473-Akt (associated with stimulation of Akt activity). We found that neither RhoA-si nor caRhoA significantly affected these phosphorylation events in C3H10T1/2 cells (Fig. S6A, B). Thus, Akt-β-catenin signaling axis is not the target of RhoA/Rock.

**Fig. 3:**
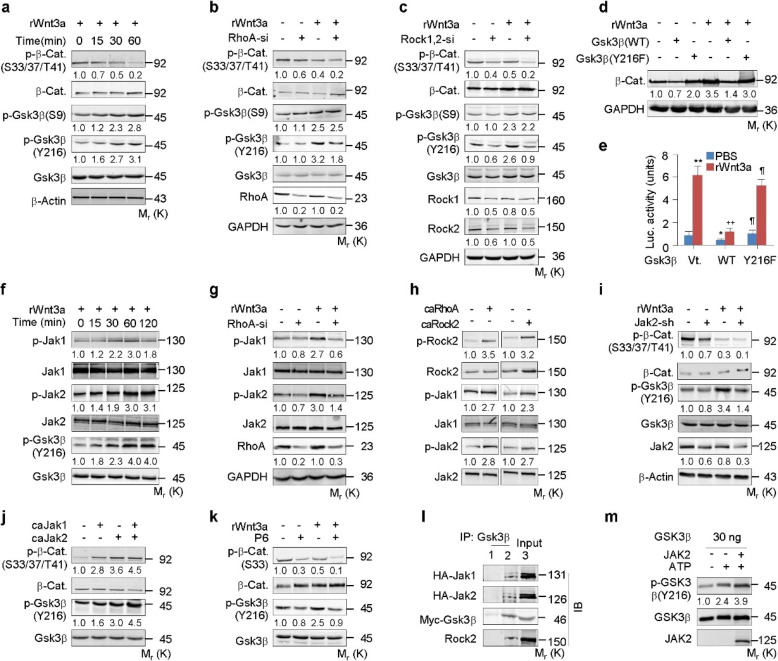
RhoA/Rock activates Jak1/2 and Gsk3β to destabilize β-catenin. **a-c** Western analyses in C3H10T1/2 cells transfected with or without RhoA-si or Rock1 + Rock2 siRNA (Rock1,2-si) and treated with or without rWnt3a at 100 ng/ml for the indicated time or 1 h. **d**, **e** Western or *Lef1-luciferase* expression analyses in C3H10T1/2 cells transfected with Gsk3β variants and treated with rWnt3a for 6 or 48 h, respectively. **f-k** Western analyses in C3H10T1/2 cells transfected with RhoA-si, caRhoA, caRock2, caJak1/2, infected with lentiviral Jak2-shRNA (Jak2-sh), or treated with P6 at 50 nM, followed by incubation with rWnt3a for the indicated times or 1 h. **l** Co-immunoprecipitation by using IgG1 or Gsk3β antibody in 293 cells transfected with HA-Jak1/2 and Myc-Gsk3β. **m** In vitro phosphorylation of GSK3β protein by active JAK2 in kinase assay buffer with or without ATP. Phosphorylated proteins were normalized to their total amounts, respectively. Mean ± SD, ^*, ¶^ *p* < 0.05, ^**, ++^*p* < 0.01, n = 4, Tukey-Kramer multiple comparisons test

We next examined the relevance of Gsk3β with antibodies specific for p-Ser33-β-catenin (a Gsk3β phosphorylation site) and p-Ser9-Gsk3β and p-Tyr216-Gsk3β (associated with inhibition and enhancement of Gsk3β activity, respectively). Wnt3a increased p-Ser9-Gsk3β or decreased p-Ser33-β-catenin in a time-dependent manner within 60 min of stimulation, but time-dependently induced an increase in p-Tyr216-Gsk3β (Fig. 3a), which is essential for full kinase activity of Gsk3β (Wu and Pan 2010; ter Haar et al. 2001). Importantly, RhoA or the combination of Rock1 and Rock2 siRNA suppressed both p-Ser33-β-catenin and p-Tyr216-Gsk3β in the basal conditions and in response to Wnt3a, without affecting p-Ser9-Gsk3β (Fig. 3b, c and Fig. S6C, D). By contrast, either caRhoA or caRock2 robustly enhanced both the p-Tyr216-Gsk3β and p-Ser33-β-catenin levels (Fig. S6E). Thus, RhoA/Rock destabilizes β-catenin while concomitantly activating Gsk3β.

We then explored the potential importance of Gsk3β phosphorylation at Tyr216 in the destabilization of β-catenin by expressing a Gsk3β variant that has the tyrosine residue mutated to phenylalanine (Y216F, phospho-null mutation) and evaluating the capacity to mediate Wnt/β-catenin signaling. In the absence of Wnt3a, the wildtype Gsk3β (WT) decreased the β-catenin levels by approximately 50% and *Lef1-luciferase* expression by approximately 60% over the vector, whereas the Y216F variant of Gsk3β caused 100% and 85% increases, respectively, over the WT levels. In the presence of Wnt3a, the WT completely abolished this induction of β-catenin levels and *Lef1-luciferase* expression, but the Y216F variant only minimally affected the induction of β-catenin levels and *Lef1-luciferase* expression (Fig. 3d, e). Thus, Gsk3β phosphorylation at Tyr216 is responsible for the RhoA/Rock-mediated Wnt/β-catenin signaling.

### Jak1/2 mediates RhoA/Rock-induced Gsk3β activation and β-catenin destabilization

Rock1/2 is a serine/threonine kinase; however, it is unlikely to be directly responsible for phosphorylation of Gsk3β at Tyr216. We explored the underlying mechanism by which RhoA/Rock induces Gsk3β phosphorylation at Tyr216 by investigating the potential relevance of the Jak tyrosine kinase that physically interacts with and is activated by Rock (Huang et al. 2013; Orgaz et al. 2014). In contrast to the relatively ubiquitous expression of Jak1 and Jak2, Jak3 expression is restricted to hematopoietic cells, vascular smooth muscle cells, and endothelium (Müller et al. 2005). We therefore examined the role of Jak1/2 in the following experiments. Western blot analyses were performed using antibodies specific for p-Tyr1022-Jak1 and p-Tyr1007-Jak2, the prerequisites for Jak1 and Jak2 activation, respectively. Concomitant with the induction of Gsk3β phosphorylation at Tyr216, Wnt3a increased both p-Jak1 and p-Jak2 over the control within 120 min of stimulation (Fig. 3f). Wnt3a robustly increased p-Jak1/2 levels in either the presence or absence of Dkk1, although Dkk1 decreased both the basal and Wnt3a-induced p-Jak1/2 levels (Fig. S7A). Likewise, the activation of Jak1 and Jak2 was essentially abolished by the expression of either a RhoA siRNA or a combination of Rock1 and Rock2 siRNA (Fig. 3g and Fig. S7B). By contrast, this activation was robustly enhanced by the expression of either caRhoA or caRock2 (Fig. 3h).

We also addressed the role of Jak1/2 in Wnt/β-catenin signaling by knockdown of Jak1/2 with specific shRNAs. Jak1-shRNA and Jak2-shRNA reduced both the p-Tyr216-Gsk3β and the p-Ser33-β-catenin levels under the basal conditions and in response to Wnt3a (Fig. 3i and Fig. S7C). By contrast, the constitutively active form of Jak1 (caJak1) or Jak2 (caJak2) apparently increased both the p-Tyr216-Gsk3β and p-Ser33-β-catenin levels (Fig. 3j). In addition, both Jak1- and Jak2-shRNAs increased the *Lef1-luciferase* expression with or without Wnt3a stimulation (Fig. S7D, E), whereas Pyridone 6 (P6), a pan-Jak inhibitor, at 50 nM, increased the *Lef1-luciferase* expression and decreased the p-Tyr216-Gsk3β and p-Ser33-β-catenin with or without Wnt3a (Fig. 3k and Fig. S7F).

We explored the potential physical interactions between Rock, Jak, and Gsk3β by co-immunoprecipitation experiments using cell lysates from C3H10T1/2 cells that co-expressed HA-tagged Jak1/2 and Myc-tagged Gsk3β. The protein complex precipitated with a Gsk3β antibody contained the exogenous HA-tagged Jak1/2 as well as the endogenous Rock2, in addition to Myc-tagged Gsk3β, as expected (Fig. 3l). The physical interaction between Jak and Gsk3β was further demonstrated by immunostaining in C3H10T1/2 cells that transiently expressed HA-Jak2 and Flag-tagged Gsk3β (Fig. S7G). We also investigated whether Gsk3β was phosphorylated at Tyr216 by Jak by in vitro phosphorylation assays using purified active human JAK2 and GSK3β. In the absence of JAK2, recombinant human GSK3β underwent more autophosphorylation at Tyr216 upon incubation with ATP. Importantly, when GSK3β was incubated with JAK2 in the presence of ATP, significantly more GSK3β phosphorylation occurred at Tyr216 (Fig. 3m). Taken together, the data indicate that RhoA/Rock activates Jak1/2 to directly phosphorylate Gsk3β at Tyr216, resulting in Gsk3β activation and β-catenin destabilization.

### Jak1/2-mediated GSK3β activation participates in osteoblastogenesis

**Fig. 4:**
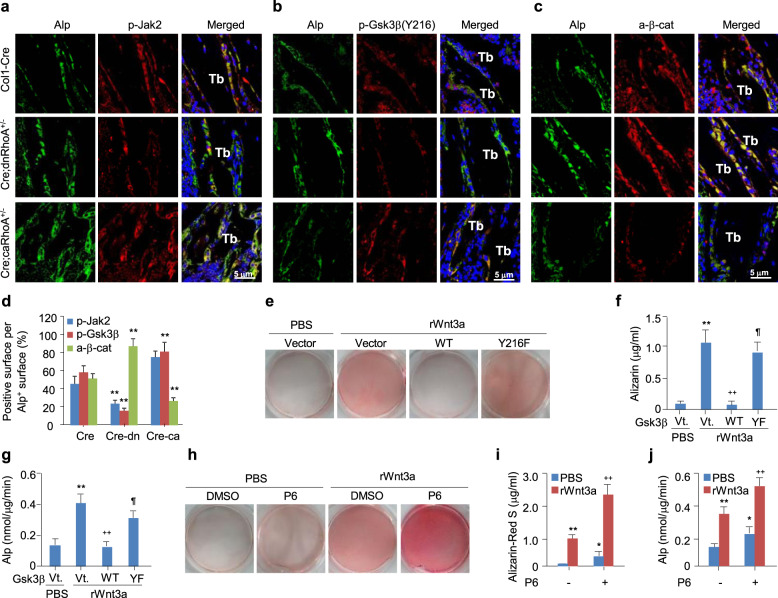
RhoA/Rock activates Jak and GSK3β to destabilize β-catenin in osteoblastic differentiation in response to Wnt3a. **a-c** Images co-stained Alp with p-Jak2, p-Tyr216-Gsk3β or active β-catenin in proximal tibia (Tb) sections of 2-month-old *Col1-Cre*, *Col1-Cre;dnRhoA*^*+/−*^, or *Col1-Cre;caRhoA*^*+/−*^ mice. **d** The percentages of p-Jak2^+^, p-Tyr216-Gsk3β^+^, or active β-catenin^+^ surface in Alp^+^ surface were quantified. Mean ± SEM, ^**^p < 0.01, *n* = 6, Tukey-Kramer multiple comparisons test. **e-j** Alizarin Red S staining and quantification or Alp activity assays in C3H10T1/2 cells infected with vector-, Gsk3β(WT)- or Gsk3β(Y216F)-expressing lentiviruses or treated with vehicle or P6 at 50 nM, and further cultured with or without 100 ng/ml of rWnt3a for 21 days or 48 h, respectively. Mean ± SD, *,^¶^ *p* < 0.05, **,^++^*p* < 0.01, *n* = 3 ~ 5, Tukey-Kramer multiple comparisons test

We confirmed a role for the Rock/Jak/Gsk3β signaling axis in regulating Wnt/β-catenin signaling by immunostaining for Alp (osteoblast marker), p-Rock2, p-Jak2, p-Tyr216-Gsk3β, and active β-catenin in longitudinal tibia sections of *Col1-Cre*, *Col1-Cre;dnRhoA*^*+/−*^, and *Col1-Cre;caRhoA*^*+/−*^ mice. Inactivation or activation of RhoA in osteoblasts was validated by detecting the numbers of p-Rock2^+^ cells in the Alp^+^ osteoblast populations (Fig. S8A, B). The numbers of p-Jak2^+^ and p-Tyr216-Gsk3β^+^ osteoblasts showed a robust decrease in tibias from *Col1-Cre;dnRhoA*^*+/−*^ mice and a robust increase in tibias from *Col1-Cre;caRhoA*^*+/−*^ mice when compared to the *Col1-Cre* mouse tibias. By contrast, the numbers of active β-catenin^+^ osteoblasts were significantly increased in the *Col1-Cre;dnRhoA*^*+/−*^ tibias and significantly decreased in the *Col1-Cre;caRhoA*^*+/−*^ tibias (Fig. 4a-d).

We investigated the physiological relevance of p-Jak and p-Tyr216-Gsk3β activation by Wnt3a using osteoblastic differentiation assays. C3H10T1/2 cells were transfected with either wild type or Gsk3β-Y216F or treated with 50 nM P6 and then examined for their ability to undergo osteoblastic differentiation in response to Wnt3a. Wildtype Gsk3β transfection reduced the Wnt3a-induced Alp activity and mineralized nodule formation by 63% and 89%, respectively, while transfection with the Y216F mutant Gsk3β decreased the Wnt3a-induced Alp activity and mineralized nodule formation by only 23% and 20%, respectively (Fig. 4e-g). Consistently, the inhibition of Jak by P6 robustly increased the Alp activity and mineralized nodule formation with or without the addition of Wnt3a (Fig. 4h-j). Thus, RhoA/Rock destabilizes β-catenin by activating Jak and Gsk3β during the regulation of osteoblastogenesis.

### RhoA loss-of-function or gain-of-function affects the bone mass in the adult mice

Overexpression of *dnRhoA* or *caRhoA* caused severe limb phenotypes in the genetic background of *Msx2-Cre;Dkk1*^*+/+*^ or *Msx2-Cre;Dkk1*^*+/−*^, respectively. However, overexpression of *dnRhoA* or *caRhoA* alone resulted in no apparent limb phenotype changes in the otherwise wild type background. This observation led us to speculate that inactivation or activation of RhoA in the wild type background underscores both the robustness of the Wnt/β-catenin signaling system and the sensitivity of the AER to a threshold level of Wnt/β-catenin signaling.

**Fig. 5:**
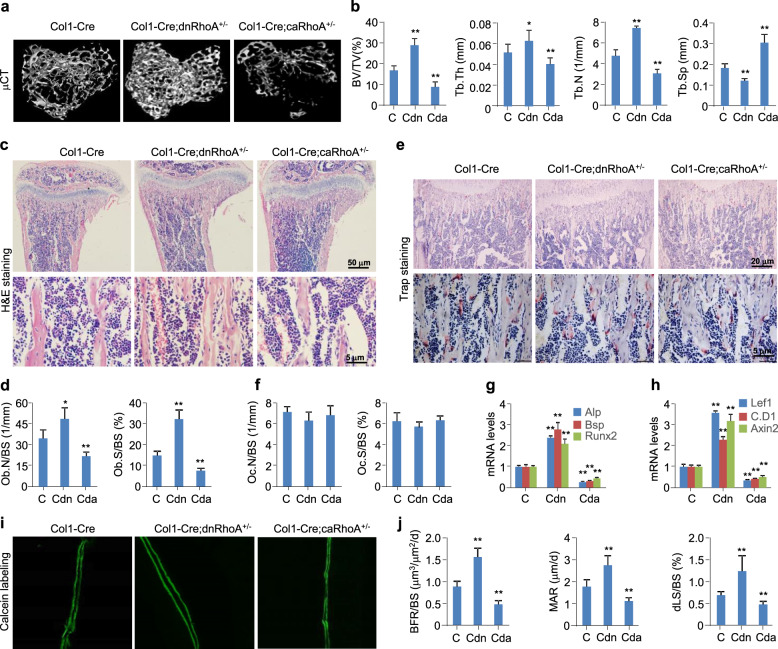
*RhoA* loss- or gain-of-function affects the bone mass. Tibias from 8-week-old male mice with the genotypes of *Col1-Cre* (C), *Col1-Cre;dnRhoA*^*+/−*^ (Cdn) or *Col1-Cre;caRhoA*^*+/−*^ (Cda) were harvested for the following analyses. **a**, **b** Representative μCT images and parameters including BV/TV, Tb.Th, Tb.N, and Tb.Sp for the proximal tibias. **c**, **d** Representative images of H&E staining and quantification of Ob.N/BS and Ob.S/BS for the proximal tibia sections. **e**, **f** Representative images of Trap staining and quantification of Oc.N/BS and Oc.S/BS for the proximal tibia sections. **g**, **h** qPCR analyses for *Alp*, *Bsp*, *Runx2*, *Lef1*, *Cyclin D1* (*C.D1*), and *Axin2* mRNA levels in femurs. **i**, **j** Dynamic bone histomorphometry and quantification for BFR/BS, MAR, and dLS/BS. Mean ± SEM, ^*^p < 0.05, ^**^p < 0.01, *n* = 8, Tukey-Kramer multiple comparisons test

We determined whether RhoA/Rock-mediated Wnt/β-catenin signaling is sufficiently robust or sensitive for regulation of bone homeostasis by generating 8-week-old male or female *Col1-Cre;dnRhoA*^*+/−*^ or *Col1-Cre;caRhoA*^*+/−*^ mice and their control littermates. Microcomputed tomography (μCT) analyses and three-dimensional reconstruction of the proximal tibias demonstrated that inactivation of RhoA in pre-osteoblasts increased the bone volume/trabecular volume (BV/TV), trabecular thickness (Tb.Th), and trabecular number (Tb.N) by 75%, 33%, and 56%, respectively, and decreased the trabecular separation (Tb.Sp) by 35%. By contrast, activation of RhoA in pre-osteoblasts decreased the BV/TV, Tb. Th, and Tb.N by 47%, 22%, and 35%, respectively, and increased the Tb.Sp by 65% (Fig. 5a, b). Hematoxylin-eosin (H&E) staining of longitudinal sections of the proximal tibias consistently revealed that inactivation of RhoA in pre-osteoblasts caused an apparent increase, while activation caused a decrease, in the bone volume, osteoblast number/bone surface (OB.N/BS), and osteoblast surface/bone surface (Ob.S/BS) (Fig. 5c, d). By contrast, tartrate-resistant acid phosphatase (Trap)-stained sections indicated that neither inactivation nor activation of RhoA in pre-osteoblasts significantly affected the parameters of osteoclast number/bone surface (Oc.N/BS) and osteoclast surface/bone surface (Oc.S/BS) in *Col1-Cre;dnRhoA*^*+/−*^ or *Col1-Cre;caRhoA*^*+/−*^ mice, respectively (Fig. 5e, f). Thus, RhoA is essential for maintaining the bone homeostasis.

Consistent with the observed alterations in bone mass, immunohistochemical staining of the proximal tibia sections indicated that inactivation of RhoA in pre-osteoblasts significantly increased, while activation decreased, the numbers of Alp^+^ osteoblasts surrounding the trabecular bone (Fig. S9A, B, D). By contrast, qPCR analyses of mRNA levels of osteoblast markers, including *Alp*, bone sialoprotein (*Bsp*), and *Runx2*, in femurs revealed that inactivation of RhoA robustly increased, while activation robustly decreased, these mRNA levels (Fig. 5g). Analyses of *Lef1*, *Cyclin D1*, and *Axin2* mRNA levels in femurs persistently indicated that inactivation of RhoA robustly increased, and activation robustly decreased, the expression of these Wnt/β-catenin signaling targets (Fig. 5h), consistent with the immunostaining of Lef1 in proximal tibia sections (Fig. S9A, C, E). Furthermore, dynamic bone histomorphometry with calcein labeling indicated that inactivation of RhoA increased the bone formation rate/bone surface (BFR/BS), mineral apposition rate (MAR), and double-labeled surface/bone surface (dLS/BS) by 82%, 55%, and 61%, respectively, whereas activation of RhoA decreased BFR/BS, MAR, and dLS/BS by 47%, 38%, and 31%, respectively (Fig. 5i, j).

Serological analyses of bone metabolism markers indicated that inactivation of RhoA in pre-osteoblasts caused a significant increase, while activation caused a significant decrease, in the levels of bone formation markers, including procollagen type 1 N-terminal propeptide (P1NP) and osteocalcin (OCN), whereas no effect was observed on the levels of bone resorption markers, including carboxyterminal collagen cross-links (CTX) and TRAP (Fig. S9F-I). Taken together, these data showed that inactivation or activation of RhoA in pre-osteoblasts affects bone formation but not bone resorption, possibly by regulating Wnt/β-catenin signaling.

### RhoA/Rock activity is inversely correlated with β-catenin signaling activity in BMMSCs from elderly human subjects

Low activity of Wnt/β-catenin signaling in BMMSCs contributes to the pathogenesis of type II osteoporosis or age-dependent bone loss (Jing et al. 2018; Manolagas and Almeida 2007). We used the bone marrow smears from young (*n* = 3, 28-, 32-, 33-year-old) or elderly (*n* = 4, 74-, 78-, 82-, and 84-year-old) male patients and the tibial sections from 5- or 10-month-old male mice, with normal bone volumes or aging-associated bone loss, respectively, to determine the potential relevance between RhoA/Rock and Wnt/β-catenin signaling. We then performed the immunostaining of p-Rock2, p-Jak1, p-Tyr216-Gsk3β, p-Ser33-β-catenin (p-β-cat, an inactive form of β-catenin), and non-p-Ser45-β-catenin (a-β-cat, an active form of β-catenin), and quantified the mRNA levels of *Lef1*, *Cyclin D1*, and *Axin2*, the targets of Wnt/β-catenin signaling, and of *Runx2*, a osteoblastic gene marker.

**Fig. 6:**
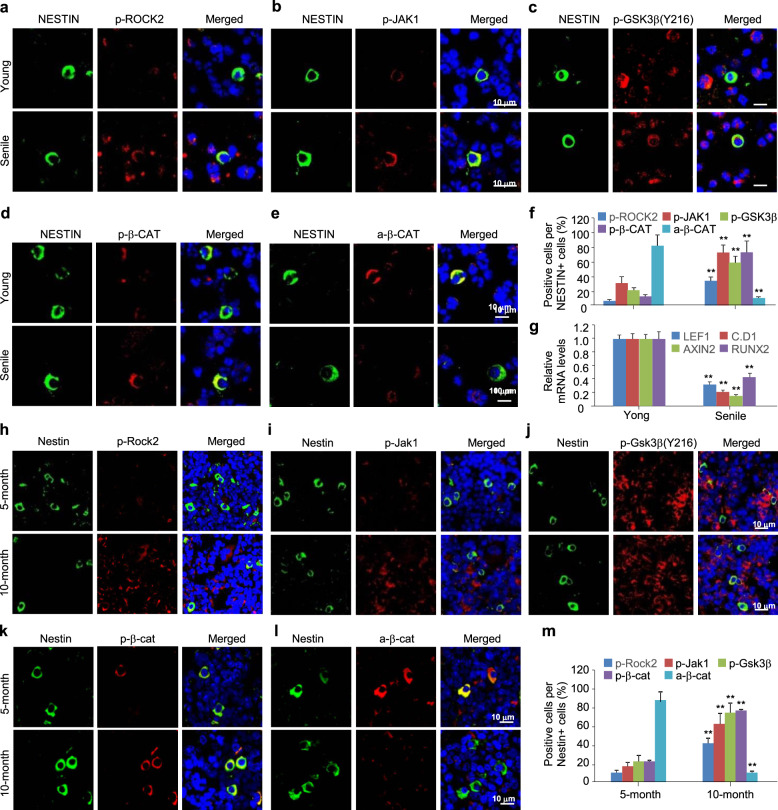
High RhoA/Rock/Jak1/Gsk3β activity is inversely correlated with Wnt/β-catenin signaling activity in the BMMSCs from elderly subjects. **a-e**, **h-l** Immunofluorescence images co-stained Nestin with p-Rock2, p-Tyr1022-Jak1, p-Tyr216-Gsk3β, p-S33-β-catenin (p-β-cat), or non-p-Ser45-β-catenin (a-β-cat) in bone marrow smears from either elderly men (n = 4) with femoral neck fractures and type II osteoporosis or young males (n = 3) with traumatic fractures and normal bone volumes, and in the proximal tibia sections of 5- or 10-month-old mice. **f**, **m** The percentages of p-Rock2^+^, p-Tyr1022-Jak1^+^, p-Tyr216-Gsk3β^+^, p-β-cat^+^, or a-β-cat^+^ cells in Nestin^+^ cells. **g** Quantitative RT-PCR analyses for *LEF1*, *Cyclin D1 (C.D1)*, *AXIN2*, and *RUNX2* mRNA levels in the BMMSCs isolated form the elderly or young men. Mean ± SEM, ^**^p < 0.01, Student’s *t* test

NESTIN-positive (NESTIN^+^) BMMSCs were scattered in the smears, and fewer NESTIN^+^ BMMSCs were obtained from the elderly men than from the young men. The immunosignals derived from p-ROCK2-, p-JAK1, p-Tyr216-GSK3β, p-β-CATENIN-, and active β-CATENIN were robustly detected, and the apparently overlapping signals between NESTIN and other molecules were readily observed in the cytoplasm of the BMMSCs (Fig. 6a-e). The NESTIN^+^ BMMSCs from the elderly men displayed considerably more p-ROCK2^+^, p-JAK1^+^, p-Tyr216-GSK3β^+^ and p-β-CATENIN^+^ cells but fewer active β-CATENIN^+^ cells than were detected in the BMMSCs from the young men (Fig. 6a-f). Consistently, considerably lower *LEF1*, *CYCLIN D1*, *AXIN2* and *RUNX2* mRNA levels were detected in the BMMSCs from the elderly men than from the young men (Fig. 6g). Likewise, Nestin^+^ BMMSCs were readily observed in the proximal tibial sections of both 5- and 10-month-old mice, although the 10-month-old mice exhibited far fewer Nestin^+^ BMMSCs. The p-Rock2-, p-Jak1, p-Tyr216-Gsk3β, p-β-catenin-, and active β-catenin-derived immunosignals were robustly detectable and the apparently overlapping signals between Nestin and other molecules were readily observed in the cytoplasm of the BMMSCs (Fig. 6h-l). The Nestin^+^ BMMSCs in the tibial sections from 10-month-old mice showed significantly more p-Rock2^+^, p-Jak1^+^, p-Tyr216-Gsk3β^+^ or p-β-catenin^+^ cells but less active β-catenin^+^ cells when compared with the BMMSCs from the 5-month-old mice (Fig. 6h-m). Thus, RhoA/Rock activation was closely correlated with the activation of Jak and Tyr216-Gsk3β and inversely correlated with the inactivation of Wnt/β-catenin signaling in the BMMSCs from elderly men.

### FZD receptors and Dkk1 coordinate RhoA/Rock activation in the regulation of β-catenin signaling in BMMSCs

**Fig. 7:**
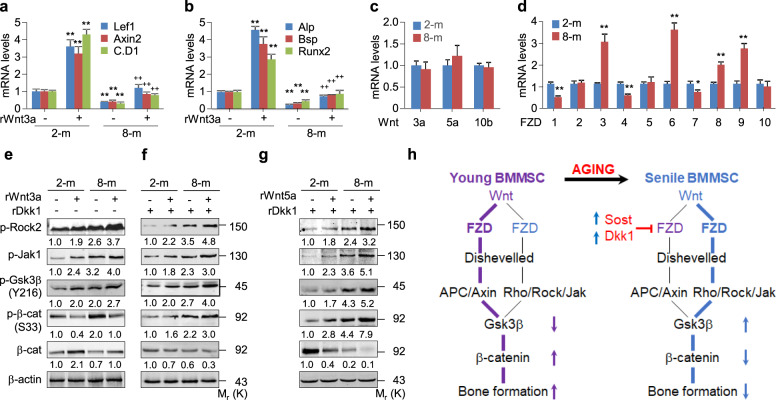
FZD receptors and Dkk1 coordinate RhoA/Rock activation to destabilize β-catenin in the BMMSCs from elderly mice. **a-d** Quantitative RT-PCR analyses for the indicated mRNA levels of BMMSCs isolated form 2- or 8-month-old mice and treated with or without rWnt3a at 100 ng/ml for 48 h. Mean ± SD, ^**^p < 0.01, n = 6, Tukey-Kramer multiple comparisons test. **e-g** Western blotting analyses in the BMMSCs isolated form 2- or 8-month-old mice and treated with or without rWnt3a/rWnt5a at 100 ng/ml in the presence or absence of rDkk1 at 100 ng/ml for 3 h. **h** An integrated working model of β-catenin signaling mediated by RhoA/Rock in the regulation of aging-associated bone loss. In the BMMSCs from young subjects, APC/Axin/Gsk3β signaling mediated by FZD1, FZD4, and FZD7 overwhelms the RhoA/Rock/Jak/Gsk3β signaling mediated by FZD3, FZD6, and FZD8 to stabilize β-catenin and, in turn, enhance bone formation. However, in the BMMSCs from elderly subjects, the activation of RhoA/Rock/Jak/Gsk3β signaling mediated by FZD3, FZD6, and FZD8, in combination with the inactivation of APC/Axin/Gsk3β signaling mediated by Dkk1, Sost, and FZD1, FZD4, and FZD7 results in the destabilization of β-catenin and the subsequent attenuation of bone formation

We compared the β-catenin signaling and osteoblastogenic activities of BMMSCs from young and elderly mice in response to Wnt3a by isolating and culturing the BMMSCs from 2- and 8-month-old mice and determining the mRNA levels of β-catenin signaling targets, including *Lef1*, *Axin2*, and *Cyclin D1*, and of osteoblastogenic markers, including *Alp*, *Bsp*, and *Runx2*. BMMSCs from two-month-old mice expressed significantly higher levels of these mRNAs than was observed in the 8-month-old BMMSCs with or without the addition of Wnt3a. However, the mRNA levels were increased more robustly in response to Wnt3a stimulation in the BMMSCs from 2-month-old mice than from 8-month-old mice (Fig. 7a, b). Thus, the BMMSCs from elderly animals have a relatively low capacity to mediate the Wnt/β-catenin signaling and osteoblastogenesis.

At present, 19 Wnt ligands and 10 FZD receptors have been identified, and the interactions between these ligands and receptors vary in a developmental and tissue-specific manner, leading to activation of β-catenin-dependent and/or β-catenin-independent Wnt signaling (Voloshanenko et al. 2017; DeBruine et al. 2017). We addressed whether the low activity of Wnt/β-catenin signaling in the BMMSCs of the elderly mice results from the alterations in Wnt ligands and/or FZD receptors by examining the mRNA levels of *Wnt3a*, *Wnt5a*, and *Wnt10b* (the representatives of Wnt ligands) and of *FZD1 ~ 10*. BMMSCs from 8-month-old mice displayed almost the same mRNA levels of *Wnt3a*, *Wnt5a*, and *Wnt10* as were found in BMMSCs from 2-month-old mice (Fig. 7c), whereas the BMMSCs from the old mice showed significantly lower mRNA levels of *FZD1*, *4*, and *7* or higher levels of *FZD3*, *6*, *8*, and *9* (Fig. 7d). FZD2, 5, 7, and 9 participate in both the β-catenin-dependent and β-catenin-independent Wnt signaling, whereas FZD3, 6, and 8 are predominantly involved in the β-catenin-independent Wnt signaling, and FZD1, 4, and 9 mainly mediate β-catenin-dependent Wnt signaling (Voloshanenko et al. 2017; DeBruine et al. 2017). Thus, the down-regulation of FZD1, 4, and 7 expression and the upregulation of FZD3, 6, and 8 expression may lead to the aberrant activation of RhoA/Rock and the low responsiveness of Wnt/β-catenin signaling in the BMMSCs from the old mice (Fig. 7h).

Wnt3a, which is capable of activating both the “canonical” and “non-canonical” Wnt signaling (Tu et al. 2007; Qu et al. 2013), decreased the p-S33-β-catenin and increased the β-catenin levels more robustly in the BMMSCs from young mice than from the old mice, though Wnt3a treatment consistently increased the p-Rock2, p-Jak1, and p-Tyr216-Gsk3β in BMMSCs from mice at both ages (Fig. 7e). Thus, Wnt3a/FZDs/RhoA/Rock/Jak-mediated Gsk3β activation counteracts the Wnt3a/FZDs/APC/Axin-mediated Gsk3β inactivation, resulting in the low responsiveness of Wnt/β-catenin signaling in the BMMSCs from the old mice (Fig. 7h). These findings are consistent with the notion that “non-canonical Wnt signaling” inhibits “canonical Wnt signaling” (Nemeth et al. 2007; Yuzugullu et al. 2009).

The high circulating levels of Dkk1 and Sost cause a significantly low activity of Wnt/β-catenin signaling in BMMSCs of patients with osteoporosis or age-associated bone loss (Coulson et al. 2017; Hampson et al. 2013). We explored the possibility that Dkk1 also functions as a molecular switch between the APC/Axin/Gsk3β signaling and the RhoA/Rock/Jak/Gsk3β signaling in controlling the β-catenin levels by western blot analyses. Unexpectedly, in the presence of Dkk1, Wnt3a treatment consistently increased the levels of p-Rock2, p-Jak1, p-Tyr216-Gsk3β, and p-S33-β-catenin but decreased the level of β-catenin in BMMSCs from mice of both ages (Fig. 7f). This suggests that Dkk1 inactivates Wnt3a/FZDs/APC/Axin/Gsk3β signaling and, in turn, activates Wnt3a/FZDs/RhoA/Rock/Jak/Gsk3β signaling, resulting in the β-catenin destabilization and bone loss (Fig. 7h).

We further addressed the role of Dkk1 by treating the BMMSCs with a combination of Dkk1 and Wnt5a, which is considered as a “non-canonical” Wnt ligand and is capable of activating RhoA/Rock (Li et al. 2019; Uehara et al. 2017). Wnt5a behaved essentially the same as Wnt3a in the presence of Dkk1, as it robustly increased the levels of p-Rock2, p-Jak1, p-Tyr216-Gsk3β, and p-S33-β-catenin and consequently destabilizes β-catenin (Fig. 7g). Thus, FZDs/APC/Axin-mediated Gsk3β inactivation surpasses the FZDs/RhoA/Rock/Jak- mediated Gsk3β activation, resulting in the β-catenin stabilization and bone formation in the BMMSCs from young mice. By contrast, FZDs/RhoA/Rock/Jak-mediated Gsk3β activation surpasses the FZDs/APC/Axin-mediated Gsk3β inactivation, resulting in the β-catenin destabilization and bone loss in the BMMSCs from old mice (Fig. 7h).

### Pharmacological inhibition of Rock antagonizes aging-associated bone loss by activating Wnt/β-catenin signaling

**Fig. 8:**
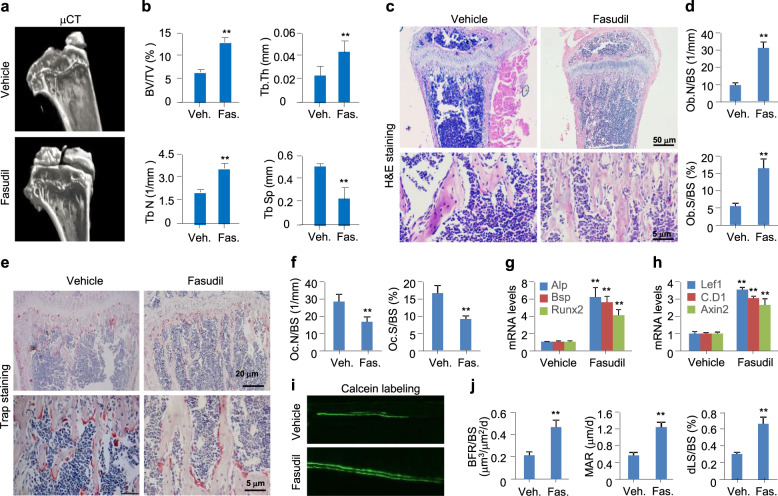
Pharmacological inhibition of Rock by Fasudil antagonizes aging-associated bone loss. 8-month-old male mice were orally administrated with vehicle (Veh.) or Fasudil (Fas.) at 100 mg/kg once daily for 2 months. Tibias were harvested for the following analyses. **a**, **b** Representative μCT images and parameters including BV/TV, Tb.Th, Tb.N, and Tb.Sp for the proximal tibias. **c**, **d** Representative images of H&E staining and quantification of Ob.N/BS and Ob.S/BS for the proximal tibia sections. **e**, **f** Representative images of Trap staining and quantification of Oc.N/BS and Oc.S/BS for the proximal tibia sections. **g**, **h** qPCR analyses for *Alp*, *Bsp*, *Runx2*, *Lef1*, *Cyclin D1 (C.D1)*, and *Axin2* mRNA levels in femurs. (**i, j**) Dynamic bone histomorphometry and quantification for BFR/BS, MAR, and dLS/BS. Mean ± SEM, ^*^p < 0.05, ^**^p < 0.01, n = 8, Tukey-Kramer multiple comparisons test

We examined whether the inhibition of RhoA/Rock is sufficient to activate Wnt/β-catenin signaling and in turn enhance the bone volume in male mice with aging-associated bone loss by assessing the pharmacological effectiveness of Fasudil, a Rock2 inhibitor, as a preventative of this bone loss. Eight-month-old male wild-type mice were orally administered with 100 mg/kg of Fasudil once daily for 2 months, and then assayed for bone volume and activity of Wnt/β-catenin signaling. At 10 months of age, the mice exhibited a significantly low trabecular bone mass, whereas Fasudil robustly preserved the mass of cancellous bone, as revealed by morphological and histological analyses (Fig. 8a, c). Three-dimensional reconstruction of the proximal tibia using μCT confirmed that Fasudil treatment at 100 mg/kg resulted in a significant increase in BV/TV, Tb.N, and Tb.Th, but a robust decrease in Tb.Sp, when compared to mice administrated the vehicle (Fig. 8b). H&E staining of longitudinal sections of the proximal tibiae consistently revealed that Fasudil treatment increased the Ob.N/BS and Ob.S/BS by 2.3- and 2.0-fold, respectively (Fig. 8c, d). By contrast, Trap-stained sections indicated that Fasudil treatment decreased the osteoclast characteristics, including Oc.N/BS and Oc.S/BS, by 41% and 45%, respectively, compared to the vehicle treatment (Fig. 8e, f).

Consistent with the increase in bone volume, Fasudil treatment significantly increased the number of Alp^+^-stained osteoblasts observed in horizontal sections of lumbar (Fig. S10A, B, D), whereas analyses of mRNA levels of osteoblast markers in femurs revealed that Fasudil treatment significantly increased the mRNA levels of *Alp*, *Bsp*, and *Runx2* (Fig. 8g). Analyses of *Lef1*, *Cyclin D1*, and *Axin2* mRNA levels in femurs indicated that Fasudil treatment also robustly increased the expression of these Wnt/β-catenin signaling targets (Fig. 8h), whereas immunostaining of Lef1 in horizontal sections of lumbar indicated that Fasudil treatment significantly increased the number of Lef1^+^-stained cells (Fig. S10A, C, E). Moreover, dynamic bone histomorphometry with calcein labeling indicated that Fasudil treatment increased the BFR/BS, MAR, and dLS/BS by 118%, 120%, and 116%, respectively (Fig. 8i, j). Serological analysis of bone metabolism markers indicated Fasudil treatment increased the levels of bone formation markers P1NP and OCN by 52% and 62%, respectively, but decreased the levels of bone resorption markers CTX and TRAP by 37% and 42%, respectively, compared to the vehicle treatment (Fig. S10F-I). Overall, these data reveal that pharmacological inhibition of RhoA/Rock prevents the aging-associated bone loss, possibly by activating Wnt/β-catenin signaling.

## Discussion

During Wnt/β-catenin signaling, Wnt3a on the one hand suppresses APC/Axin/Gsk3β to stabilize β-catenin (Wu et al. 2008), but on the other hand activates RhoA/Rock to destabilize β-catenin by activating Jak and Gsk3β (Fig. 7h). Thus, RhoA/Rock activation constitutes a negative regulatory mechanism that allows fine-tuning the amplitude of β-catenin signaling.

The limb phenotypes resulting from *RhoA* loss-of-function or gain-of-function in the AER can also arise in the presence, rather than the absence, of the *Dkk1* transgene. This suggests that *RhoA* loss-of function or gain-of-function in the wildtype background underscores both the robustness of the Wnt/β-catenin signaling system and the sensitivity of the AER to a threshold level of Wnt signaling. Thus, under physiological conditions, the β-catenin stabilization mediated by APC/Axin/GSK3β signaling overwhelms the destabilization mediated by RhoA/Rock/Jak/Gsk3β signaling to regulate the limb outgrowth. By contrast, *RhoA* loss-of-function or gain-of-function in pre-osteoblasts is sufficient to cause the bone phenotypes, and aberrant activation of RhoA/Rock in aging-associated bone loss causes the inactivation of β-catenin in the BMMSCs from elderly subjects. Thus, under pathological conditions, such as type II osteoporosis, the β-catenin destabilization mediated by RhoA/Rock/Jak/Gsk3β signaling overwhelms the β-catenin stabilization mediated by APC/Axin/GSK3β signaling to regulate the bone volume.

Fasudil, an inhibitor of Rock2, has been reported to induce osteoblastic differentiation by promoting BMP2 expression (Kanazawa et al. 2010). Here, we present an alternative mechanism wherein Fasudil antagonizes aging-associated bone loss. Both the previous and our present studies demonstrate a therapeutic potential for Fasudil as a preventative treatment for osteoporosis (Shimokawa and Takeshita 2005). Likewise, AZD2858, a Gsk3 inhibitor, significantly increases the bone mass in rats by upregulating the β-catenin levels (Marsell et al. 2012), further validating this inhibitor in the osteoporosis therapy by targeting the ROCK/JAK/GSK3β signaling.

Although RhoA-mediated Wnt/β-catenin signaling has been identified by the previous works (Ordóñez-Morán et al. 2008; Rodrigues et al. 2014; Miyagi et al. 2015; Kim et al. 2017; Rossol-Allison et al. 2009) and in the present study, a role of RhoA-mediated β-catenin-independent Wnt signaling has also been established for the osteogenic differentiation in response to mechanical stimulation (Shi et al. 2012; Li et al. 2015; Li et al. 2019). In addition, RhoA mediates Wnt5a/Ror2 signaling to improve the actin ring formation in osteoclasts and to facilitate bone resorption (Uehara et al. 2017). Thus, these findings support the notion that the actual role of RhoA/Rock in β-catenin-dependent or β-catenin-independent Wnt signaling depends on the duration, degree and the cellular and tissue context.

How GSK3β is modulated and fine-tuned by Wnt to stabilize β-catenin remains a fundamental question in the field. We suggest that RhoA/Rock activates Jak by directly phosphorylating Jak at Tyr216, consistent with the Jak activation by Wnt3a (Fragoso et al. 2012; Hao et al. 2006). Generally, Jak is recruited from the cytoplasm to bind and activate cytokine receptors upon the cytokine stimulation. Here, we suggest that cytoplasmic Jak is recruited into the protein complex consisting of Rock and Gsk3β during Wnt signaling.

One recent study has indicated that Rock1 physically interacts with and activates Jak2 in response to leptin (Huang et al. 2013). However, the detailed mechanism by which RhoA/Rock regulates Jak during Wnt signaling is unknown and warrants further study. Although emerging evidences emphasizes the importance of Jak/Stat pathways in bone development and metabolism (Li 2013), the findings presented here suggest that Jak activation destabilizes β-catenin and prevents the maintenance of bone volume, consistent with the finding that constitutive Jak2 activation in mesenchymal cells promotes adipogenesis and reduces osteogenesis in the bone marrow (Yue et al. 2016).

Jak inhibitors are approved as treatments for rheumatoid arthritis, however, the role of Jak/Stat inhibition in bone homeostasis is not fully understood. Ruxolitinib, an inhibitor of Jak/Stat, decreases osteoblastic differentiation of hMSCs in vitro and reduces ectopic bone formation in vivo (AlMuraikhi et al. 2018). However, these findings appear to be at odds with our present finding that P6, a pan-JAK inhibitor, stabilizes β-catenin and promotes osteoblast differentiation. They also differ from a recent study that showed that the Jak inhibitors tofacitinib and baricitinib significantly increase osteoblast function and bone mass by stabilizing β-catenin in vivo, with no direct effects on osteoclasts in vitro (Adam et al. 2020). These discrepancies could be explained by the fact that the interactions among JAKs and STATs occur in a developmental and tissue-specific manner and that JAK/STAT signaling pathways have diverse effects in different cell contexts (Murray 2007; Gadina et al. 2018).

In conclusion, we identify RhoA/Rock/Jak/Gsk3β(Tyr216) as a hitherto uncharacterized signaling cascade that acts primarily to destabilize β-catenin. Thus, this study highlights a precise regulation of Wnt signaling and provides an additional mechanism for regulation of limb development, while also identifying a potential new therapeutic target for aging-associated bone loss.

### Supplementary Information

**Additional file 1.**

**Additional file 2.**

## Data Availability

Dataset described in this work can be downloaded from the supplementary Figs. S1–10 and Tables S1–3 from the journal website. Materials request should be addressed to the corresponding author.

## Abbreviations

BMMSCs: Bone marrow-derived mesenchymal stromal cells
FZD: Frizzled
Lrp5/6: Low-density lipoprotein receptor-related protein 5 or 6
Dvl: Dishevelled
Gsk3β: Glycogen synthase kinase-3β
APC: Adenomatous polyposis coli
Lef1: Lymphoid enhancer-binding factor 1
(Tcf): T cell factors
Dkk1: Dickkopf 1
PCP: Planar cell polarity
CE: Convergent extension
JNK2: c-Jun N-terminal kinase 2
Rock: Rho-kinase
Jak: Janus kinase
β-catenin: β-cat
PMCOBs: Primary murine calvarial osteoblasts
dnRhoA: Dominant negative form of RhoA
caRhoA: Constitutively active form of RhoA
Col1: Collagen1α1
caRock2: Constitutively active form of Rock2
Alp: Alkaline phosphatase
Akt: Protein kinase B
WT: Wild type
P6: Pyridone 6
AER: Apical ectodermal ridge
Fgf8: Fibroblast growth factor 8
Bmp4: Bone morphogenetic protein 4
μCT: Microcomputer tomography
BV/TV: Bone volume/trabecular volume
Tb.Th: Trabecular thickness
Tb.N: Trabecular number
Tb.Sp: Trabecular separation
Ob.N/BS: Osteoblast number/bone surface
Ob.S/BS: Osteoblast surface/bone surface
Trap: Tartrate-resistant acid phosphatase
Oc.N/BS: Osteoclast number/bone surface
Oc.S/BS: Osteoclast surface/bone surface
BFR/BS: Bone formation rate/bone surface
MAR: Mineral apposition rate
dLS/BS: Double-labeled surface/bone surface
Bsp: Bone sialoprotein
P1NP: Procollagen type 1 N-terminal propeptide
OCN: Osteocalcin
CTX: Carboxyterminal collagen cross-links
STR: Short tandem repeat
FBS: Fetal bovine serum
GAPDH: Glyceraldehyde-3-phosphate dehydrogenase
β-GP: β-glycerophosphate.
