# Correction: RhoA/Rock activation represents a new mechanism for inactivating Wnt/β-catenin signaling in the aging-associated bone loss

**DOI:** 10.1186/s13619-023-00185-4

**Published:** 2024-02-10

**Authors:** Wei Shi, Chengyun Xu, Ying Gong, Jirong Wang, Qianlei Ren, Ziyi Yan, Liu Mei, Chao Tang, Xing Ji, Xinhua Hu, Meiyu Qv, Musaddique Hussain, Ling-Hui Zeng, Ximei Wu

**Affiliations:** 1 Department of Pharmacology, Zhejiang University School of Medicine, 866 Yuhangtang Road, Hangzhou, 310058 China; 2 Department of Biology and Genetics, University of North Carolina-Chapel Hill, Chapel Hill, NC 27599 USA; 3 Department of Orthopeadic Surgery of Sir Run Run Shaw Hospital, Zhejiang University School of Medicine, Hangzhou, 310016 China; 4 Department of Pharmacology, Zhejiang University City College, 51 Huzhou Street, Hangzhou, 310015 China; 5 Translational Research Program in Pediatric Orthopaedics, The Children’s Hospital of Philadelphia, Philadelphia, PA 19104 USA

**Correction: Cell Regen 10, 8 (2021)**

**https://doi.org/10.1186/s13619-020-00071-3**

Following publication of the original article (Shi et al. 2021), the authors have identified errors in Figs. 1d and 3i which occurred during the figure assembly process. The β-actin bands in Fig. 1d were mistakenly compiled from similar experiments in a previous publication by the same group (Gong et al. 2014), conducted within the same time frame as the experiments in Fig. 1d. To address this, the authors made corrections in Fig. 1d in this revision. Furthermore, the β-catenin band in Fig. 3a was inadvertently reused in Fig. 3i, and the new Fig. 3 containing the correct β-catenin in Fig. 3i has been provided below.

**Fig. 1:**
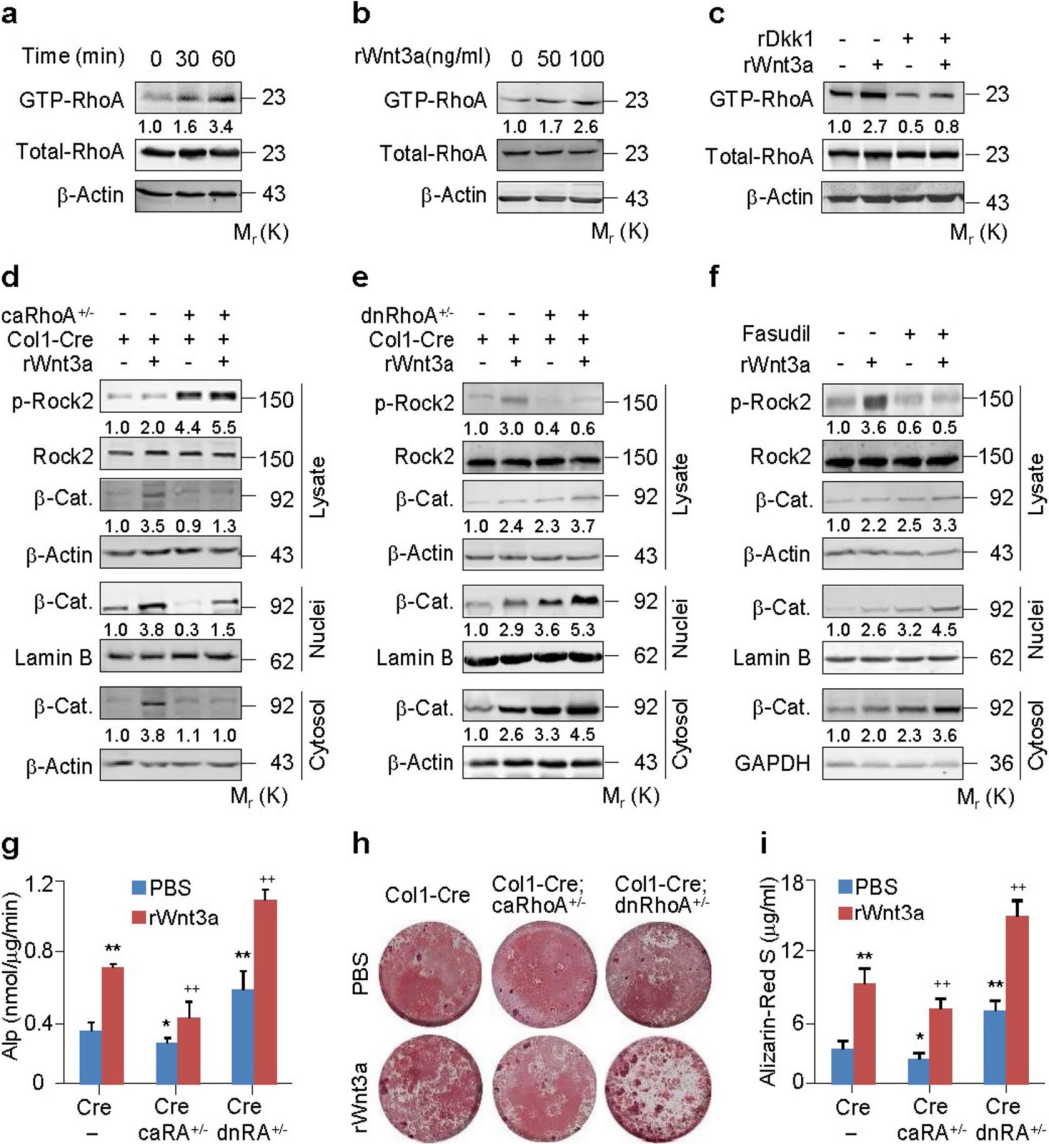
RhoA/Rock constraints Wnt/β-catenin signaling and osteoblastic differentiation. **a-c** RhoA activation assays in primary murine calvarial osteoblasts (PMCOBs) stimulated with rWnt3a at 100 ng/ml or the indicated concentrations for the indicated times or 60 min in the presence or absence of recombinant Dkk1 (rDkk1) at 100 ng/ml. **d**,**e** Western analyses of β-catenin in cytosolic and nuclear fractions of PMCOBs with the indicated genotypes of *Col1-Cre* (*Cre*), *Col1-Cre;caRhoA* ^*+*^ */* ^*−*^ (*Cre;caRhoA* ^*+*^ */* ^*−*^) or *Col1-Cre;dnRhoA* ^*+*^ */* ^*−*^ (*Cre;dnRhoA* ^*+*^ */* ^*−*^), and in the presence or absence of rWnt3a for 3 h. **f** Western analyses of β-catenin (β-cat) in cytosolic and nuclear fractions of PMCOBs treated with or without Fasudil at 20 μM and stimulated with or without rWnt3a for 3 h. **g-i** Alp activity and mineralization nodule formation assays and their quantification in PMCOBs with the indicated genotypes and stimulated with or without rWnt3a at 100 ng/ml for 48 h and 21 d, respectively. Mean ± SEM, ^*^p < 0.05, ^**,++^ *p* < 0.01, *n* = 4, Tukey–Kramer multiple comparisons test

**Fig. 3:**
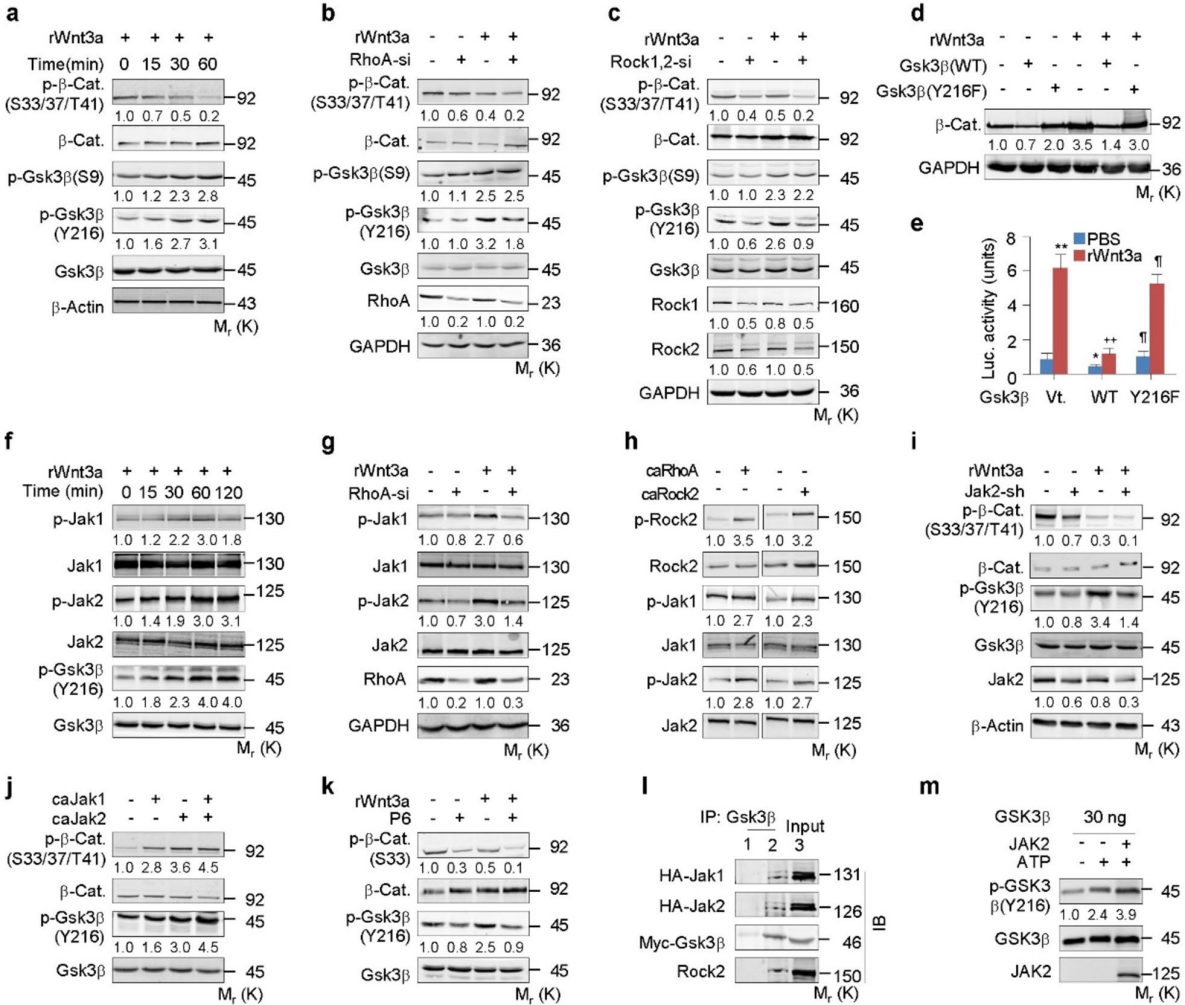
RhoA/Rock activates Jak1/2 and Gsk3β to destabilize β-catenin. **a-c** Western analyses in C3H10T1/2 cells transfected with or without RhoA-si or Rock1 + Rock2 siRNA (Rock1,2-si) and treated with or without rWnt3a at 100 ng/ml for the indicated time or 1 h. **d**, **e** Western or *Lef1-luciferase* expression analyses in C3H10T1/2 cells transfected with Gsk3β variants and treated with rWnt3a for 6 or 48 h, respectively. **f-k** Western analyses in C3H10T1/2 cells transfected with RhoA-si, caRhoA, caRock2, caJak1/2, infected with lentiviral Jak2-shRNA (Jak2-sh), or treated with P6 at 50 nM, followed by incubation with rWnt3a for the indicated times or 1 h. **l** Co-immunoprecipitation by using IgG1 or Gsk3β antibody in 293 cells transfected with HA-Jak1/2 and Myc-Gsk3β. **m** In vitro phosphorylation of GSK3β protein by active JAK2 in kinase assay buffer with or without ATP. Phosphorylated proteins were normalized to their total amounts, respectively. Mean ± SD, ^*, ¶^ p < 0.05, ^**,++^ *p* < 0.01, n = 4, Tukey–Kramer multiple comparisons test

It's important to note that despite these corrections, all the results and conclusions in this article remain consistent and unaffected. The authors deeply regret any inconvenience caused by these errors and sincerely apologize for them.

The original article (Shi et al. 2021) has been corrected.
