# Supplementary material for: RhoA/Rock activation represents a new mechanism for inactivating Wnt/β-catenin signaling in the aging-associated bone loss
Source: Cell Regen. 2021 Mar 3;10:8. doi: 10.1186/s13619-020-00071-3 (PMC7925793; doi:10.1186/s13619-020-00071-3)
Supplement: Supplementary file 1 — Additional file 1. [file 13619_2020_71_MOESM1_ESM.zip › Supplementary Figure legendsR1.docx]

**RhoA/Rock activation represents a new mechanism for inactivating Wnt/β-catenin signaling in the aging-associated bone loss**

**Supplementary Figure legends**

**Figure S1.** RhoA, Rac1, and Cdc42 activation in response to Wnt3a. (**A-C**) RhoA activation assays in C3H10T1/2, HEK293T, and L929 cells treated the indicated dosage of rWnt3a for 1h. (**D-F**) Rac1, Cdc42 or RhoA activation assays in C3H10T1/2 cells infected with RhoA-, Cdc42-, or Rac1-shRNA-expressing lentiviruses and stimulated with or without rWnt3a at 100 ng/ml for 1h. GTP-RhoA, -Cdc42 or -Rac1 was normalized to total RhoA, Cdc42 or Rac1, respectively.

**Figure S2.** Activation or inactivation of RhoA suppresses or enhances Wnt/β-catenin signaling, respectively. (**A-C**) Quantitative RT-PCR analyses for *Lef1* and *Cyclin D1* (*C.D1*) mRNA levels in PMCOBs isolated from neonates with the indicated genotypes or from wild-type neonates treated with Fasudil at 20 μM, and stimulated with or without rWnt3a at 100 ng/ml for 48 h. (**D, E**) Western and *Lef1-luciferase* reporter assays in C3H10T1/2 cells transfected with constitutively active form of RhoA (caRhoA) or RhoA siRNA (RhoA-si) and cultured with or without rWnt3a at 100 ng/ml for 48 h. Mean ± SD, **p*<0.05, **,^++^*p*<0.01, n=3-6, Tukey–Kramer multiple comparisons test.

**Figure S3.** Rock1/2 behaves the same as RhoA in regulating the Wnt/β-catenin signaling. (**A**) Western and *Lef1-luciferase* reporter assays in C3H10T1/2 cells transfected with Rock1-si, Rock2-si, or their combination, and cultured with or without rWnt3a for 48 h. (**B, C**) *Lef1-luciferase* reporter assays in C3H10T1/2 cells treated with Y27632 at indicated dosages or transfected with caRock2, and further cultured with or without rWnt3a for 48 h. Mean ± SD, **p*<0.05, **,^++^*p*<0.01, n=3-6, Tukey–Kramer multiple comparisons test.

**Figure S4.** RhoA regulates the limb outgrowth upon the presence of β-catenin. Whole-mount staining of skeletons and limbs from E16.5 embryos with the indicated genotypes. FL: forelimb, HL: hindlimb, n=4-6.

**Figure S5.** *RhoA* loss- or gain-of-function regulates the size of limb bud and the targets of Wnt/β-catenin signaling in AER. (**A**) H&E and TUNEL staining in E10.5 forelimb buds with the indicated genotypes. (**B**) Whole-mount in situ hybridization of *FGF8* and *BMP4* in E10.5 forelimb buds with the indicated genotypes. (**C, D**) Quantification of the TUNEL staining and Whole-mount in situ hybridization, respectively. Ventral view for all limb buds, anterior to the lower and posterior to the upper. White or red dot lines separate the apical ectodermal ridge (AER) from the zone of polarizing activity (ZPA). Mean ± SEM, *,^¶^ *p*<0.05, **,^++^*p*<0.01, n=4-6, Tukey–Kramer multiple comparisons test..

**Figure S6.** RhoA/Rock regulates the activity of β-catenin, Akt, and Gsk3β. (**A, B**) Western analyses of p-β-catenin(Ser552), p-Akt(Ser473), β-catenin, Akt, and RhoA in C3H10T1/2 cells transfected with RhoA siRNA or caRhoA, and cultured with or without rWnt3a at 100 ng/ml for 1 h. (**C-E**) Western analyses of p-β-catenin(Ser33), p-Gsk3β(Ser9, Tyr216), β-catenin, Gsk3β, Rock1, Rock2, p-Rock2, and GAPDH in C3H10T1/2 cells transfected with Rock1 siRNA, Rock2 siRNA, caRhoA or caRock2, and cultured with or without rWnt3a at 100 ng/ml for 1 h.

**Figure S7.** Jak1/2 is involved in the Wnt/β-catenin signaling. (**A**) Jak1 and Jak2 activation assays in C3H10T1/2 cells treated with or without rDkk1 (100ng/ml) and rWnt3a (100ng/ml) for 1 h, rDkk1 was added 3 h before and during the culture with rWnt3a. (**B**) Western analyses of p-Jak1/2 versus total Jak1/2 in C3H10T1/2 cells transfected with the combination of Rock1 and 2 siRNA (Rock1,2-si) and cultured with or without rWnt3a for 1 h. (**C**) Western analyses of p-β-catenin, p-Gsk3β, and total Jak1 and Gsk3β and β-catenin in C3H10T1/2 cells infected with scramble- or Jak1-shRNA-expressing lentiviruses and cultured with or without rWnt3a for 1h. (**D-F**) *Lef1-luciferase* expression in C3H10T1/2 cells infected with scramble-, Jak1- or Jak2-shRNA lentiviruses or treated with vehicle or P6 at 50 nM, and cultured with or without rWnt3a for 48 h. Mean ± SD, *,^+^*p*<0.05, **,^++^*p*<0.01, n=5, Tukey–Kramer multiple comparisons test. (**G**) Immunofluorescence staining using Flag and HA antibodies in C3H10T1/2 cells transiently expressing Flag-Gsk3β and HA-Jak2 and cultured with or without rWnt3a for 1 h.

**Figure S8.** Validation of RhoA inactivation and activation in osteoblasts of *Col1-Cre;dnRhoA^+/-^* and *Col1-Cre;caRhoA^+/-^* mice, respectively. (**A, B**) Representative images co-stained Alp with IgG or p-Rock2 in proximal tibia sections of 2-month-old male *Col1-Cre*, *Col1-Cre;dnRhoA^+/-^*,or *Col1-Cre;caRhoA^+/-^* mice and their quantification. IgG was used as the negative control. Mean ± SEM, ***p*<0.01, n=6, Tukey-Kramer multiple comparisons test.

**Figure S9.** *RhoA* loss- or gain-of-function of regulates the bone mass. Tibias and sera from 8-week-old male mice with the indicated genotypes were harvested for the following analyses. (**A-C**) Representative images of H&E staining and immunohistological staining of Alp and Lef1 in tibia sections. (**D, E**) Quantification of Alp^+^ or Lef1^+^ surfaces per bone surfaces. (**F-I**) ELISA determination for serum bone formation and resorption markers including P1NP, OCN, CTX, and TRAP. Mean ± SEM, ^*^*p*<0.05, ^**^*p*<0.01, n=8, Tukey–Kramer multiple comparisons test.

**Figure S10.** Pharmacological inhibition of Rock2 by Fasudil restores the aging-associated bone loss. 8-month-old male mice were orally administrated with vehicle (Veh.) or Fasudil (Fas.) at 100 mg/kg, once daily, for 2 months, and lumbar and serum were harvested for the following assays. (**A-C**) Representative images of H&E staining and immunohistological staining of Alp and Lef1 in lumbar sections. (**D, E**) Quantification of Alp^+^ or Lef1^+^ surfaces per bone surfaces. (**F-I**) ELISA determination of serum bone formation and resorption markers including P1NP, OCN, CTX, and TRAP. Mean ± SEM, ^**^*p*<0.01, n=8, Tukey–Kramer multiple comparisons test.
