# Supplementary material for: RhoA/Rock activation represents a new mechanism for inactivating Wnt/β-catenin signaling in the aging-associated bone loss
Source: Cell Regen. 2021 Mar 3;10:8. doi: 10.1186/s13619-020-00071-3 (PMC7925793; doi:10.1186/s13619-020-00071-3)
Supplement: Supplementary file 2 — Additional file 2. [file 13619_2020_71_MOESM2_ESM.docx]

| **Table S1. Primers for amplifying the target genes** | | |
| --- | --- | --- |
|  | **Sense(5'---3')** | **Antisense(5'---3')** |
| mouse GSK3β | CGGCGGCCGCAATGTCGGGGCGACCGAGAACCA(NotⅠ) | CCGGTACCTCAGGTGGAGTTGGAAGCTGAT(KpnⅠ) |
| human β-catenin | CGGGATCCATGGCTACTCAAGCTGATTTGAT(BamHⅠ) | CCGGTACCTTACAGGTCAGTATCAAACCAGG(KpnⅠ) |
| mouse dominant-active ROCK2 | CGGGATCCCCGACGGGGAAAATGCCCGGCG(BamHⅠ) | GCCTCGAGTTAGGATATCTGAGAGCTCTGGTTC(XhoⅠ) |
| mouse dominant-negative ROCK2 | CGGGATCCAAAGAGAAGATCATGAAAGAGC(BamHⅠ) | GCCTCGAGTTAGCTTGGCTTGTTTGGAGCA(XhoⅠ) |
| mouse JAK1 | CGGCGGCCGCAATGCAGTATCTAAATATAAAAGA(NotⅠ) | CCGGTACCTTATTTTAAAAGTGCTTCAAAT(KpnⅠ) |
| mouse JAK2 | CGGGATCCATGGGAATGGCCTGCCTTACAAT(BamHⅠ) | CGGCGGCCGCTCACGCAGCTATACTGTCCCGGA(NotⅠ) |
| mouse GSK3β Y216F | ATTTATCTGTTCTCGGTACTACA(TAT→TTT) | GAAACATTGGGCTCTCCTCGGA |
| mouse dominant-active JAK1(V658F) | TTTCCGAGATGTGGAAAATATC(GTC→TTC) | CACACGCCGTAGAGGTACACTA |
| mouse dominant-active JAK2(V617F) | TTTCTGTGGAGAGGAGAACAT(GTC→TTC) | CAGACACCATAATTCAAAACCA |

| **Table S2. Gene-specific siRNAs** | | |
| --- | --- | --- |
|  | **Sense(5'---3')** | **Antisense(5'---3')** |
| mouse ROCK1-1# | CAGCAAAUUUGAAAUGAUUAAdtdt | UUAAUCAUUUCAAAUUUGCUGdtdt |
| mouse ROCK1-2# | CAAGAAGUAAAUGAACAUAAAdtdt | UUUAUGUUCAUUUACUUCUUGdtdt |
| mouse ROCK2-1# | UUGGAUAAACAUGGACAUCUAdtdt | UAGAUGUCCAUGUUUAUCCAAdtdt |
| mouse ROCK2-2# | CAGCGGAAAGCUGAUCAUGAAdtdt | UUCAUGAUCAGCUUUCCGCUGdtdt |
| mouse RhoA-1# | CGCGUCUGCCAUGAUUGGUUAdtdt | UAACCAAUCAUGGCAGACGCGdtdt |
| mouse RhoA-2# | UCAGCCCUAUAUAUCAUUCUAdtdt | UAGAAUGAUAUAUAGGGCUGAdtdt |

| **Table S3. Oligoneucleotides for generation of lentiviral shRNAs** | | |
| --- | --- | --- |
|  | **Sense(5'---3')** | **Antisense(5'---3')** |
| mouse Daam1-1# | AACGGGAAAGAGCAGGCAGAGAATTCAAGAGATTCTCTGCCTGCTCTTTCCCTTTTTTC | TCGAGAAAAAAGGGAAAGAGCAGGCAGAGAATCTCTTGAATTCTCTGCCTGCTCTTTCCCGTT |
| mouse Daam1-2# | AACGGAAGAGGAGGAGAGGAGTATTCAAGAGATACTCCTCTCCTCCTCTTCCTTTTTTC | TCGAGAAAAAAGGAAGAGGAGGAGAGGAGTATCTCTTGAATACTCCTCTCCTCCTCTTCCGTT |
| mouse Gαq11-1# | AACGGGAAGTGGATCCATTGCTTTTCAAGAGAAAGCAATGGATCCACTTCCCTTTTTTC | TCGAGAAAAAAGGGAAGTGGATCCATTGCTTTCTCTTGAAAAGCAATGGATCCACTTCCCGTT |
| mouse Gαq11-2# | AACGCCAAGTTGGTGTACCAGAATTCAAGAGATTCTGGTACACCAACTTGGCTTTTTTC | TCGAGAAAAAAGCCAAGTTGGTGTACCAGAATCTCTTGAATTCTGGTACACCAACTTGGCGTT |
| mouse Dvl2-1# | AACGGCTTTCTTCGTACACCTATTTCAAGAGAATAGGTGTACGAAGAAAGCCTTTTTTC | TCGAGAAAAAAGGCTTTCTTCGTACACCTATTCTCTTGAAATAGGTGTACGAAGAAAGCCGTT |
| mouse Dvl2-2# | AACGGGAAGAGAUCUCGGACGACTTCAAGAGAGTCGTCCGGTCTCTTCCCTTTTTTC | TCGAGAAAAAAGGGAAGAGAUCUCGGACGACTCTCTTGAAGTCGTCCGGTCTCTTCCCGTT |
| mouse JAK1-1# | AACGGAAAATGAATTGAGTCGATTTCAAGAGAATCGACTCAATTCATTTTCCTTTTTTC | TCGAGAAAAAAGGAAAATGAATTGAGTCGATTCTCTTGAAATCGACTCAATTCATTTTCCGTT |
| mouse JAK1-2# | AACGGAAATCACCCACATTGTAATTCAAGAGATTACAATGTGGGTGATTTCCTTTTTTC | TCGAGAAAAAAGGAAATCACCCACATTGTAATCTCTTGAATTACAATGTGGGTGATTTCCGTT |
| mouse JAK2-1# | AACGGCAAACCAGGAATGCTCAATTCAAGAGATTGAGCATTCCTGGTTTGCCTTTTTTC | TCGAGAAAAAAGGCAAACCAGGAATGCTCAATCTCTTGAATTGAGCATTCCTGGTTTGCCGTT |
| mouse JAK2-2# | AACGGGAATGGCCTGCCTTACAATTCAAGAGATTGTAAGGCAGGCCATTCCCTTTTTTC | TCGAGAAAAAAGGGAATGGCCTGCCTTACAATCTCTTGAATTGTAAGGCAGGCCATTCCCGTT |
